# Simulating the hydrologic cycle in coal mining subsidence areas with a distributed hydrologic model

**DOI:** 10.1038/srep39983

**Published:** 2017-01-20

**Authors:** Jianhua Wang, Chuiyu Lu, Qingyan Sun, Weihua Xiao, Guoliang Cao, Hui Li, Lingjia Yan, Bo Zhang

**Affiliations:** 1 State Key Laboratory of Simulation and Regulation of Water Cycles in River Basins, China Institute of Water Resources and Hydropower Research, Beijing, 100038, P. R. China

## Abstract

Large-scale ground subsidence caused by coal mining and subsequent water-filling leads to serious environmental problems and economic losses, especially in plains with a high phreatic water level. Clarifying the hydrologic cycle in subsidence areas has important practical value for environmental remediation, and provides a scientific basis for water resource development and utilisation of the subsidence areas. Here we present a simulation approach to describe interactions between subsidence area water (SW) and several hydrologic factors from the River-Subsidence-Groundwater Model (RSGM), which is developed based on the distributed hydrologic model. Analysis of water balance shows that the recharge of SW from groundwater only accounts for a small fraction of the total water source, due to weak groundwater flow in the plain. The interaction between SW and groundwater has an obvious annual cycle. The SW basically performs as a net source of groundwater in the wet season, and a net sink for groundwater in the dry season. The results show there is an average 905.34 million m^3^ per year of water available through the Huainan coal mining subsidence areas (HCMSs). If these subsidence areas can be integrated into water resource planning, the increasingly precarious water supply infrastructure will be strengthened.

Coal is the main energy source in China, accounting for about 70% of primary energy production and consumption^1^,^2^. Coal is also one of the most important energy sources in the world, and its exploitation and utilisation are increasing year by year^3^,^4^. The consumption of coal plays an important role in the economy^5^,^6^. However, coal mining is often accompanied by environmental pollution and landscape destruction^7^,^8^,^9^,^10^, of which land surface subsidence due to underground coal mining is one of the direct causes^11^,^12^,^13^,^14^,^15^,^16^. While the remediation of destruction caused by open pit coal mining has been the subject of many studies^17^,^18^,^19^,^20^, the remediation of coal mining subsidence is a rare subject globally, except in China. The surface subsidence caused by underground coal mining in the densely populated plain of China has a great impact on the economy, society, environment and ecology^21^,^22^, and so studies on land reclamation and ecological restoration of subsidence regions are abundant^21^,^23^,^24^,^25^,^26^. In addition, ground subsidence in the plain area has significantly changed the topography and formed several depressions, which may intercept and store water from precipitation as their volumes increase. Because of this, it has been suggested to exploit subsidence depressions as plain reservoirs for water storage and supply, providing a new way to utilise coal mining subsidence^27^,^28^.

The development and management of coal mining subsidence, from the perspective of water resource management, requires evaluation of the quantities and cyclicity of the water resources of subsidence depressions. However, water recharge and discharge in subsidence areas are complicated processes, which involve multiple uncertain factors such as precipitation, evaporation, flow from surrounding rivers, and groundwater. It is difficult to explain the mechanisms of the hydrologic cycle of artificial depressions; for example, the source of water in subsidence areas and its relationship to the exchange between surface water and groundwater, and the response of overland flow and underground flow impacted by ground subsidence, are all complex. It should be noted that the current foundation of quantitative research on the water balance of subsidence areas is quite weak, and the relevant literature is relatively sparse. Moreover, no research on the dynamic processes of subsidence area water within the basin hydrologic cycle has yet been found, which increases the difficulty of our research.

The aim of this study is to quantify the water exchange mechanisms of coal mining subsidence areas, including the accumulation of water and the evaluation of water resource potential. For this purpose, using the distributed hydrologic model is a necessary method. However, clarifying the complex interactions between surface water and groundwater is a major problem. To address this, we used a distributed hydrologic model, MODCYCLE^29^,^30^,^31^,^32^ as the basis for this study. The model’s embedded three-dimensional numerical simulation module for groundwater can solve the general problem of the combined simulation of surface water and groundwater. Many other models can also be used to simulate the interaction between surface water and groundwater, such as MODFLOW^33^, MIKE-SHE^34^, and SWATMOD^35^. Considering the similarity of subsidence areas and other surface waters, these models may be capable of the hydrologic simulation of the subsidence areas. In this research the MODCYCLE was modified specifically to simulate the water balance and water level dynamics in the subsidence areas.

In this study, the model was developed by coupling it with the River-Subsidence-Groundwater Model (RSGM), according to the hydrologic characteristics of typical subsidence areas located in the plain with a high groundwater table. The results provide data on the development of typical subsidence areas, which may be used as reference data for the remediation of other subsidence areas.

## Results

**Figure 1:**
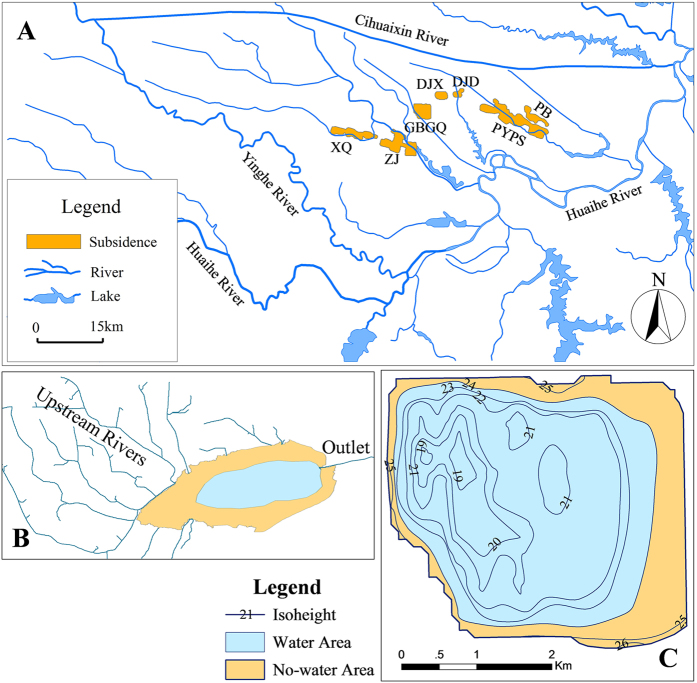
Spatial distribution of the HCMS (**A**). The subsidence areas including XQ, ZJ, GBGQ, DJX, DJD, PYPS and PB, are distributed in a region surrounded by the Huaihe, Yinghe and Cihuaixin Rivers, which is taken as the study area for the water exchange simulation (area 4012 km^2^). (**B**) is a horizontal schematic diagram of an open subsidence area. (**C**) shows the ground surface isoheights and the water area on December 1st, 2009, in the GBGQ subsidence area, which is a closed subsidence area. Open and closed subsidence areas are explicated in Table 1. Maps were created using ArcGIS 9.3 (http://www.esri.com/software/arcgis/arcgis-for-desktop).

Of the many subsidence areas in China, the Huainan coal mining subsidence areas (HCMSs), located in the middle plain of Huaihe River Basin, were selected as a typical case for this study. The HCMSs cover wide areas (total 108.3 km^2^), subside deeply (the maximum depth is 7.6 m), and have shallow groundwater depth (around 1.5 m). These features make the water budget of the subsidence area rather complex, and significantly influence the agriculture and ecology of the surrounding environment. Figure 1A illustrates the spatial distribution of the HCMSs. Table 1 shows some features of the HCMSs, and the hydraulic connections between rivers or lakes and each subsidence area. Other information about the HCMS and the study area can be found in Supplementary Information 1.

Informed by the results from modelling the hydrologic cycle of the study area from 2001 to 2010, we analysed the accumulation mechanism of water in a typical closed subsidence area, and evaluated the water resource formation across all subsidence areas.

### Mechanism for the accumulation of water in a typical closed subsidence area

All the subsidence areas store abundant water, and the water level changes with the surrounding groundwater table. Due to the high groundwater table and the abundant precipitation in the study area, it is intuitively believed that the water in the open subsidence areas derives from groundwater, precipitation and upstream runoff, and the water in the closed subsidence areas derives from groundwater and precipitation. However, it is difficult to quantify the contribution from the groundwater using the conventional method. Therefore, we selected the GBGQ subsidence area, which has not yet been disturbed by stream flow and human water diversion, as a typical case to study the source of the water in a subsidence area. The ground surface isoheights and the water area of the GBGQ subsidence area on December 1st, 2009 are shown in Fig. 1(C).

**Figure 2:**
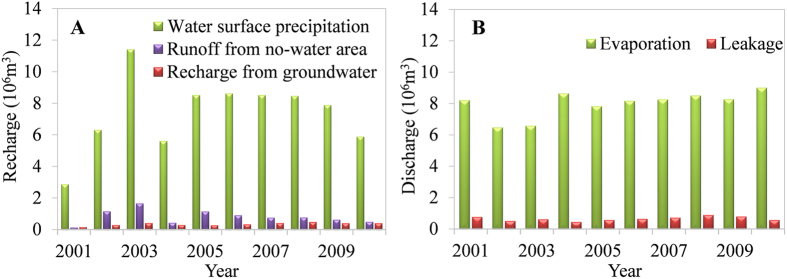
Annual components of recharge (**A**) and discharge (**B**) of the GBGQ subsidence area, simulated from 2001 to 2010.

Figure 2 illustrates the simulated annual recharge (A) and discharge (B) of the GBGQ subsidence area from 2001 to 2010. Rainfall received by the water surface of the subsidence area accounts for the majority of the recharge, followed by the runoff from the no-water areas in the subsidence range, and the recharge from groundwater accounts for the least. Water in the subsidence area is mainly removed by evaporation, with only a little water leaking into groundwater.

Analysing the annual average values of the water budget of the GBGQ, the recharge component from water surface precipitation accounts for 86.6%, runoff from no-water areas 9.4%, and the remainder (4.0%) consists of inputs from groundwater under water areas and under no-water areas in the subsidence areas. The proportion of evaporation from the subsidence area is 92.5% of the total discharge, the remainder (7.5%) leaking into groundwater. The analysis of the water budget shows that the influence of precipitation and evaporation on the water balance of the closed subsidence area is much greater than that of groundwater.

**Figure 3:**
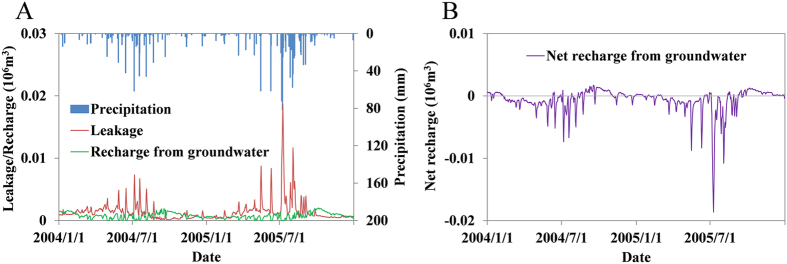
The daily process (**A**) of precipitation, leakage, and recharge from groundwater for the GBGQ subsidence area in 2004–2005, which is typical of the whole simulated period. There is a positive correlation between precipitation and leakage. Ordinarily, in the wet season recharge is less than leakage, and in the dry season recharge is greater than leakage. The difference between the two fluxes is the net recharge from groundwater for the GBGQ, as shown in (**B**).

In order to further explore the exchange mechanisms involved, we selected the daily data for recharge from groundwater and leakage into groundwater during 2004‒2005, to analyse the water flux between the aquifer and the GBGQ, as shown in Fig. 3(A). Precipitation dominates the water recharge into the GBGQ, so the daily precipitation is also shown in the illustration. The leaking of subsidence area water into the groundwater mainly occurs in the wet season, which shows that there is a significant positive correlation between precipitation and leakage, and the linear correlation coefficient is 0.54. There is a negative correlation between recharge from groundwater and precipitation.

The variability of groundwater–subsidence area water fluxes is directly decided by the difference between the groundwater table and the subsidence area water level. Water may move from a higher level to a lower level at any time and any locality. The daily net recharge process of the subsidence area was calculated by subtracting the leakage from the recharge for every day during 2004‒2005, as illustrated in Fig. 3(B). When the net recharge in one day is positive, it indicates that the water recharge is greater than the water leakage for the GBGQ on that day; otherwise, the leakage is greater than the recharge.

The water budget of the GBGQ subsidence area shows that the interactions between the groundwater and the subsidence area water have an obvious annual cycle. The subsidence area water basically performs as a net leakage into groundwater in the wet season, and a net recharge from groundwater in the dry season. The mechanism is that concentrated, high frequency precipitation in the wet season can raise the subsidence area water level in a short time, whereas the groundwater table rises slowly due to the hysteresis effect of groundwater recharge; and so at this stage, the subsidence area water leaks into the groundwater. In the dry season, the subsidence area receives little precipitation, which results in a substantial reduction of water recharge. However, the evaporation does not decrease like the rainfall. In this case, the subsidence area water level falls faster than the groundwater table, and the subsidence area is recharged by the groundwater.

### Simulation evaluation of water exchange and water resource formation in the HCMSs from 2003‒2010

The total capacity of the HCMSs is 267.3 million m^3^, but divided into several subsidence areas, most of which are connected with the surrounding rivers and lakes, and integrated into the hydrologic cycle of the study area. Therefore the water resources of open subsidence areas are not only limited by the precipitation, surface runoff and groundwater discharge within the subsidence area, but also by the flow from upstream.

Due to the ZJ and PYPS subsidence spreading to nearby natural lakes and forming contiguous depressions with them, the simulation and evaluation are carried out using the whole of these depressions, including both the subsidence areas and the lakes. The whole of these depressions are referred to as subsidence areas. During the simulation period, 2001‒2002 was the spin-up period of the model; therefore we only present the annual average recharges, discharges and storage changes from 2003‒2010, as shown in Table 2.

The annual average precipitation in the study area from 2003‒2010 was 1,022 mm, which is 134 mm more than the annual average value of the long series precipitation (1954–2010). Under this condition, the total water recharge of all subsidence areas in the study area is 1,313.0 million m^3^ per year, of which the flow from upstream accounts for 1,174.0 million m^3^, precipitation (WSP and RNWA) about 106.6 million m^3^, and recharge from groundwater about 32.5 million m^3^. Therefore, with the present drainage pattern of the study area, most of the recharge derives from upstream of the subsidence areas. In terms of the discharge from the subsidence areas, water drainage accounts for 86.6% of the total, with other outputs including evaporation and leakage accounting for less than 15%. Discharges also include artificial diversion. Water is mainly pumped from lakes which have a hydraulic relationship with some subsidence areas, but not directly from the subsidence areas.

Analysis of the water balance can be used to evaluate the water resources of the subsidence areas. Note that the subsidence areas XQ and ZJ are located in the same catchment, and have an upstream-downstream relationship. The drainage from the XQ subsidence area will flow into the ZJ subsidence area, so the volume repeated between them should be deducted. Therefore the drainage of the upstream subsidence area is subtracted from the inflow of the downstream subsidence area. The process for calculating the water resource quantity in each subsidence area is shown in Table 3.

As an annual average, the total water resource of the HCMSs is about 905.3 million m^3^, of which the water resources of the open subsidence areas are about 892.5 million m^3^, which is far greater than the 12.9 million m^3^ of the closed subsidence areas. Thus the runoff from outside the subsidence areas is an important source of water recharge in the HCMSs. As the 74.5 million m^3^ of water surface evaporation is unavailable, the total water resource of the HCMSs is about 830.8 million m^3^.

Analysing the composition of the water resource, the flow from upstream is the largest source, accounting for 84.6% of the total. The groundwater only contributes a little to the water resource of the HCMSs, at 3.6%. Regardless of whether the subsidence areas have hydraulic contact with rivers, groundwater is never the major source of the subsidence area water, even in the plain area with its high groundwater table.

## Discussion

The results of a typical subsidence analysis show that the recharge from groundwater accounts for only about 4.0% of the total recharge, the rest being from precipitation. A closed subsidence area is filled by water gradually, as the coal mining subsidence expands. Once there is water in a subsidence area, the main factor controlling the recharge is not groundwater seepage into the subsidence area, but precipitation and evaporation. Data shows that the average annual precipitation is close to the water surface evaporation in the study area. Even if there is no groundwater seepage into the mining subsidence area, and only the precipitation and runoff inside the subsidence area, the closed subsidence area can still maintain a water area of about 71% of the subsidence area. The ratio of the water area to the subsidence area may increase along with the ratio of precipitation to evaporation, which is in agreement with the study of Mao and Fang^36^ on subsidence area water in Yantenglianghuai, where the HCMS is located.

The mutual exchange between the subsidence area water and the groundwater is driven by the difference in water levels, rather than unilaterally depending on the phreatic water table. The interaction process between the groundwater and the subsidence area water has an obvious annual cycle. The water in the subsidence area basically performs as a net leakage into the groundwater in wet seasons, and a net recharge from the groundwater in dry seasons, which can result in a fine-tuning of the subsidence area water quantity. Subsidence areas on plains, like the HCMSs, have high groundwater tables, which can prevent the leakage of water from the coal mining subsidence area, so that the water in the subsidence area can be stored for a long time.

The total maximum capacity of the HCMS reaches 267.3 million m^3^, and averages 1313.0 million m^3^ of water stored and drained through the subsidence depressions each year from 2003 to 2010. Taking into account the repeated volumes from different subsidence depressions in the same basin, there are 905.3 million m^3^ per year of water available for use. If these subsidence areas can be incorporated into water projects for water resource control and utilisation, the increasingly insecure water supply situation will be somewhat relieved.

## Methods

To study the mechanism by which water accumulates in coal mining subsidence areas, and to assess the water resource availability in these areas, we simulated the hydrologic cycle of the study area using the MODCYCLE model, which couples a hydrologic model with a groundwater flow model. Here we give a brief introduction to the principle, construction and calibration of the model.

### Model principle

#### Governing equation for the water balance of the subsidence areas

The RSGM is a secondary development, based on the distributed hydrologic model MODCYCLE, which is a surface water and groundwater coupling simulation model that has been applied several times successfully^30^,^32^,^37^,^38^,^39^,^40^,^41^,^42^. In the limited space available here, we only introduce the main principle of the RSGM. A general framework structure, the interaction between hydrology and groundwater in MODCYCLE, and other theory about the RSGM are presented in Supplementary Information 2.

Generally, a subsidence area may receive flow from multiple upstream rivers, but drain through a single outlet. The subsidence area is divided into a water area and a no-water area, as shown in Fig. 1(B). The model assumes that the water level at every position in the subsidence area is identical at any point in time. The water area and the no-water area are in a dynamic relationship. Each area depends on the change of the water’s edge, and the sum of the water area and the no-water area equals the area of the subsidence area (see Equation 1).


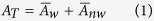


where *A*_*T*_ is the total area of the subsidence, constant during the simulation (L^2^), and 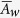 and 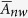 are the water area and no-water area respectively (L^2^).

For a subsidence area, the water balance in an arbitrary period can be described by:


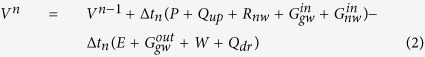


**Figure 4:**
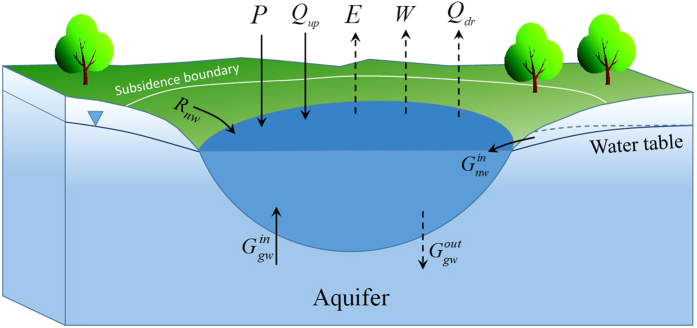
Profile schematic diagram of the subsidence area and its water budget fluxes. Map was created using PowerPoint 2013 (https://products.office.com/en-us/powerpoint).

There are 9 water fluxes associated with the water balance, shown in Fig. 4, in which *n* represents the *n*th period, *V*^*n*^ and *V*^*n*−1^ are the water volumes of the subsidence area at the end and the beginning of the *n*th period respectively (L^3^), Δ*t*_*n*_ is the time step (a daily time step is adopted in our simulation, i.e. Δ*t*_*n*_ = 1 (T)), *P, Q*_*up*_, *R*_*nw*_, 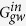, and 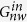 are the water area precipitation, flow from upstream, no-water area runoff, recharge from the groundwater under the water area, and recharge from the groundwater under the no-water area in each time step (L^3^T^−1^); and *E*, 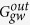, *W*, and *Q*_*dr*_ are water area evaporation, leakage into the aquifer, water withdrawal, and drainage through the outlet from the subsidence area water in each time step, respectively (L^3^T^−1^).

#### Spatial discretisation

**Figure 5:**
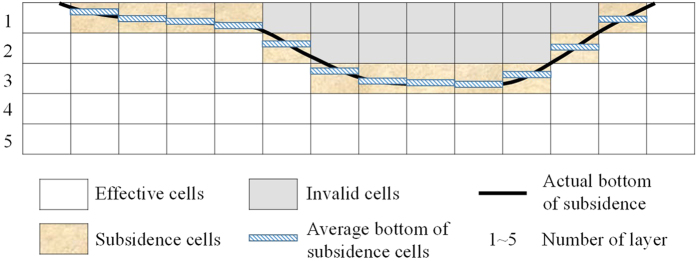
The schematic profile of the discrete aquifer and the handling of subsidence cells’ elevation. Map was created using PowerPoint 2013 (https://products.office.com/en-us/powerpoint).

Due to the interaction between the subsidence area water and the groundwater (Equation 2), the sub-items of the water balance generally need to be calculated by a groundwater numerical method, which requires spatial discretisation. The pre-subsidence aquifer of the study area was discretised according to a finite-difference grid. The schematic profile of the discrete aquifer is shown in Fig. 5. After the ground surface collapses, partial cells change to invalid cells. Those cells along the bottom of the subsidence area are the subsidence cells, whose elevations are not real. Therefore we modified the elevations of the subsidence cells with the actual bottom, by averaging the corresponding subsidence elevation.

#### Simulation of conversion between subsidence area water and groundwater

The levels of the subsidence area water and groundwater vary continuously. When the subsidence area water level is higher than the groundwater table, the subsidence area water is a source of groundwater; and conversely it is a groundwater sink when it is lower. The flux *Q* between them is calculated by the Darcy formula using the infiltration area:


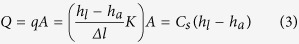


where *q* and *A* are the infiltration intensity (LT^−1^) and infiltration area (L^2^) respectively between the subsidence and the adjacent aquifer, *h*_*l*_ and *h*_*a*_ are the levels of the subsidence area water and groundwater respectively (L), Δ*l* is the seepage path between *h*_*l*_ and *h*_*a*_ (L), *K* is the saturated hydraulic conductivity (LT^−1^), and *C*_*s*_ = *KA*/Δ*l*, is the transmissivity (L^2^T^−1^).

### Model building

The main work involved in building the model is the data processing for the model database, including spatial data and hydrologic cycle data, and their processing. Taking into account the data demand of the model, and the data available, we built the model using meteorological and hydrologic data from 2001 to 2010. The main spatial data and hydrologic data are shown in Table 4. Partial data and its processing for MODCYCLE are shown in Supplementary Information 3.

In our study, the aquifer was subdivided into two layers, a shallow aquifer and a deep aquifer with variable thickness. Each aquifer consisted of 132 rows and 276 columns of uniformly spaced model cells, which are 500 m × 500 m according to the finite-difference method. Each layer has 36,432 cells, of which 16,166 cells are in the study area and the others are invalid in the simulation.

### Model calibration

The built model should be calibrated to make sure it has the capacity to simulate the actual hydrologic cycle in the study area. In this section we carry out parameter calibration by comparing the measured data and the simulated output. In the collected data, several measured series of surface water level and groundwater table are available. Artificial calibration was adopted to calibrate the parameters of the model. The measured surface water level data was used to calibrate the hydrologic cycle process, and the measured groundwater table data was used to calibrate the groundwater process. Sensitive parameters adjusted in the model calibration, and their ranges, are shown in Table 5.

Uncertainty is common in hydrologic models at present. It may take a lot of effort to complete an uncertainty analysis of the model parameters, so we did not specially carry out this work. We adjusted some key sensitive parameters into reasonable ranges by a manual method, to narrow the range of parameters as far as possible. By this method, the 95% confidence interval of simulated values, calculated by MODCYCLE using every parameter combination in the above ranges, may cover most of the measured points.

#### Comparison of measured and simulated surface water levels

**Figure 6:**
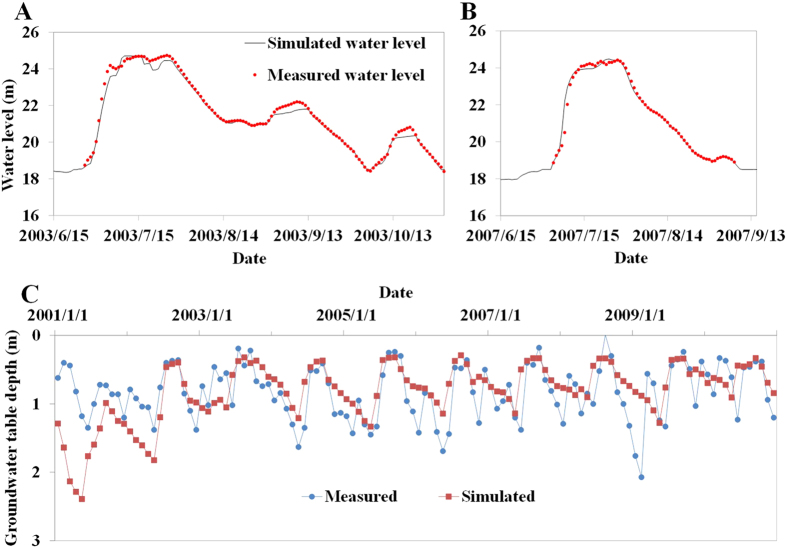
Comparison between simulated values and measured values for water level during flood seasons in 2003 (**A**) and 2007 (**B**); and comparison between simulated and measured depth of the groundwater table in Yangcunji Well from 2001 to 2010 (**C**).

As the study area lacks hydrologic stations, there is no river flow data, but only some temporary observation data for water levels in flood periods in 2003 and 2007 in the lower reaches of the ZJ subsidence area. The observation point and ZJ subsidence area are adjacent, so the data can be used to test the model in simulating surface runoff and subsidence area water levels. The results of calibration (2003 and 2007) indicate that the model can reflect the measured water level, as shown in Fig. 6(A and B).

#### Comparison of measured and simulated groundwater tables

There are six shallow groundwater observation wells in the study area, with a measurement interval of 5 days. Due to space limitations, here we only show the contrast between simulated and measured groundwater tables at the Yangcunji Well (Fig. 6(C)). The comparison results for the other five wells can be found in Supplementary Information 4. The model cannot simulate the groundwater table depth of a well because of the spatial scale limitation. It can only calculate the average value within a certain region (i.e. one grid cell of the groundwater model). Therefore, it is difficult to obtain a precise fit with the measured processes of the groundwater table, but it is still possible to study the groundwater table fluctuations from the periodicity and amplitude.

In brief, the results of the model calibration prove that the River-Subsidence-Groundwater Model basically reflects the actual hydrologic cycle of the study area from 2003 to 2010, and indicate that this method is effective for the study of the accumulation mechanism of subsidence area water, and evaluation of water resources flowing into the HCMS.

## Additional Information

**How to cite this article**: Wang, J. *et al*. Simulating the hydrologic cycle in coal mining subsidence areas with a distributed hydrologic model. *Sci. Rep.* **7**, 39983; doi: 10.1038/srep39983 (2017).

**Publisher's note:** Springer Nature remains neutral with regard to jurisdictional claims in published maps and institutional affiliations.

## Supplementary Material

Supplemental Information

## Figures and Tables

**Table 1:** The description of each subsidence area.

Subsidence	Lowest elevation (m)	Average elevation of sideline(m)	Area (hm^2^)	Maximum capacity (10^6^ m^3^)	Hydraulic connection with river or lake
XQ	17.0	23.4	1,633	46.8	open
ZJ	18.0	24.4	2,440	61.9	open
GBGQ	19.3	24.6	1,257	28.1	closed
DJX	21.0	23.0	469	5.7	closed
DJD	21.0	22.4	331	1.2	closed
PYPS	14.0	22.0	3,833	111.8	open
PB	17.8	22.1	867	11.8	open

The open subsidence areas have direct hydraulic connection with nearby rivers or lakes; the closed subsidence areas have no direct hydraulic connection with nearby rivers or lakes.

**Table 2:** The annual average water budget of every subsidence area from 2003‒2010 (10^6^ m^3^).

Subsidence area	Recharge					Discharge					Storage change		
	WSP	RNWA	RG	FUP	Subtotal	WSE	Leakage	Water withdrawal	Drainage	Subtotal	Storage in early 2003	Storage in late 2010	AC
XQ	10.5	1.5	3.2	393.2	408.4	10.2	1.6	0.0	396.5	408.4	26.4	26.4	0.0
ZJ	25.5	12.3	21.1	550.5	609.3	21.4	5.8	48.0	534.1	609.3	49.6	49.6	0.0
GBGQ	8.1	0.8	0.4	0.0	9.3	8.2	0.7	0.0	0.0	8.8	12.8	16.9	0.5
DJX	1.5	0.8	0.3	0.0	2.7	1.5	0.4	0.0	0.8	2.7	0.7	0.4	0.0
DJD	0.0	0.8	0.1	0.0	0.9	0.0	0.0	0.0	0.9	0.9	0.0	0.0	0.0
PYPS	31.2	9.3	6.7	221.0	268.1	30.4	2.8	41.5	193.3	268.1	41.2	41.2	0.0
PB	2.9	1.4	0.8	9.3	14.4	2.9	0.3	0.0	11.2	14.4	4.1	4.1	0.0
**Total**	**79.6**	**27.0**	**32.5**	**1,174.0**	**1,313.0**	**74.5**	**11.7**	**89.6**	**1,136.8**	**1,312.6**	**134.8**	**138.7**	**0.5**

Water surface precipitation (WSP), runoff from no-water area (RNWA), recharge from groundwater (RG, RGWA and RGNWA) and flow from upstream (FUP) are the recharge fluxes; and water surface evaporation (WSE), leakage, water withdrawal and drainage are the discharge fluxes. Annual average storage change (AC) is calculated as the difference between the storage in early 2003 and the storage in late 2010 divided by 8 years.

**Table 3:** Evaluation of the annual average water resources for the subsidence areas from 2003‒2010.

Subsidence area	Source of water			(4)	(5)	Water resource quantity
	(1)	(2)	(3)			
	WSP and RNWA	RG	FUP	Drainage	FUP subtracting repeated volume	
XQ	12.0	3.2	393.2	396.5	393.2	408.3
ZJ	37.7	21.1	550.5	534.1	154.0	212.8
GBGQ	9.0	0.4	0.0	0.0	0.0	9.3
DJX	2.3	0.3	0.0	0.8	0.0	2.7
DJD	0.9	0.1	0.0	0.9	0.0	0.9
PYPS	40.5	6.7	221.0	193.3	209.8	257.0
PB	4.3	0.8	9.3	11.2	9.3	14.4
**Total**	**106.6**	**32.5**	**1,174.0**	**1,136.8**	**766.3**	**905.3**

The water resource quantities are calculated as (1)+(2)+(3)+(5). (Unit 10^6^ m^3^). WSP and RNWA are water surface precipitation and runoff from no-water areas, RG is recharge from groundwater, FUP is flow from upstream, and ‘FUP subtracting repeated volume’ represents the FUP minus the drainage of the upstream subsidence area. For instance, the FUP subtracting repeated volume of ZJ is its FUP minus the drainage of XQ, which is its upstream subsidence area. PB is the upstream subsidence area to PYPS.

**Table 4:** Main data for the model.

Category	Data	Source/Description
Basic geographic information	Digital elevation model (DEM)	Cell size 90 m × 90 m
	Digital river map	1:250,000
	Distribution map of land use and cover	1:100,000 (2005)
	Distribution map of soil type	1:1,000,000
	Subsidence data	China University of Mining and Technology
Weather information	Precipitation, temperature, wind speed, solar radiation, relative humidity, and location distribution	National Weather Service and local hydrology bureau
Soil parameters	Hydraulic conductivity, porosity, density, field capacity, and soil water supply capacity	China soil scientific database
Hydrogeological information	Aquifer distribution, bottom elevation of shallow aquifers, aquifer thickness, transmissivity, etc.	Local hydrogeology survey report
Crop information	Crop growth period and parameters	Field investigation
Water project information	Water project parameters	Local water resources investigation and evaluation reports and planning reports
Groundwater information	Groundwater observation wells and observation series of buried depth	Local hydrology bureau and Huainan Mining Industry (Group) Co., Ltd.
Water use information	Agricultural irrigation, urban industrial and domestic water use, and rural water use	Local water resources bulletin

**Table 5:** Sensitive parameters adjusted in the model calibration and their range for MODCYCLE.

Simulation process	Key parameter	Explanation	Range
Hydrologic cycle simulation	SOL_AWC	Available water capacity of soil layers, the difference between field capacity water content and water content at wilting point (mm).	0.05‒0.20
	SOL_K	Saturated hydraulic conductivity of soil layers (mm/hr).	0.05‒12.0
	ESCO	Soil evaporation compensation coefficient.	0.92‒1.0
	EPCO	Plant uptake compensation factor.	0.95‒1.0
	SURLAG	Surface runoff lag coefficient.	3.0‒7.0
	GWDMN	Threshold water level in shallow aquifer for base flow to occur (m).	1.0‒6.0
	ALPHA_BF	Baseflow alpha factor.	0‒1.0
Groundwater flow simulation	TRAN	Transmissivity along rows (m^2^/s).	85‒300
	VCONT	Vertical hydraulic conductivity divided by the thickness from a layer to the layer below (s^−1^).	0‒0.00016
	SC1	Primary storage coefficient.	0.005‒0.037
Subsidence area water simulation	SED_K	Saturated hydraulic conductivity of subsidence bottom sediment (m/day).	0.0005‒0.005
	EVWBCOF	Correction coefficient for evaporation from water surface.	0.8‒1.0
	RNFCN	SCS curve number of no water area in subsidence.	30‒100
	PCPRCHCOF	Ratio of precipitation recharging into groundwater to total precipitation	0.15‒0.50
