# Supplementary material for: Simulating the hydrologic cycle in coal mining subsidence areas with a distributed hydrologic model
Source: Sci Rep. 2017 Jan 20;7:39983. doi: 10.1038/srep39983 (PMC5247746; doi:10.1038/srep39983)
Supplement: Supplemental Information [file srep39983-s1.pdf]

Supplemental Information for

Simulating the hydrologic cycle in coal mining subsidence areas with a distributed hydrologic model

Jianhua Wang, Chuiyu Lu*, Qingyan Sun, Weihua Xiao, Guoliang Cao, Hui Li, Lingjia Yan, Bo Zhang

State Key Laboratory of Simulation and Regulation of Water Cycles in River Basins, China Institute of Water Resources and Hydropower Research, Beijing 100038, China

Supplementary Information 1: Background of the Huainan coal mining subsidence areas (HCMSs)

Huainan coal seams, with an area of about 3,000 km^2, are distributed in the middle region and spread across the mainstream of the Huaihe River Basin (HRB). The coal seams located in the north side of the mainstream are buried below the ground surface 1,000 ~ 1,200 m and include 9 to 18 layers; and the total average thickness of minable seams are 20–30m. Longwall top coal caving (LTCC) method is used for extraction of mining of thick seams[1]. Collapses caused by deep coal mining spread up to the soft stratum, and lead to ground subsidence (Fig. S1).

Ground subsidence causes damage to land resources, river systems, water facilities, and residential buildings, changes the original landscapes (as shown in Fig. S2), and may modify the original hydrologic cycle processes. The main purpose of this study is to investigate the hydrologic cycle in the Huainan coal mining subsidence areas (HCMSs), including processes associated with rainfall, runoff, overland flow, river flow, evaporation, infiltration and other processes occurring in basin scale. As the subsidence area is just a part of the river basin, it is necessary to investigate the impacts of subsidence on hydrologic processes in basin scale. In this study, the basin area containing all subsidence areas is included into the modeling domain.

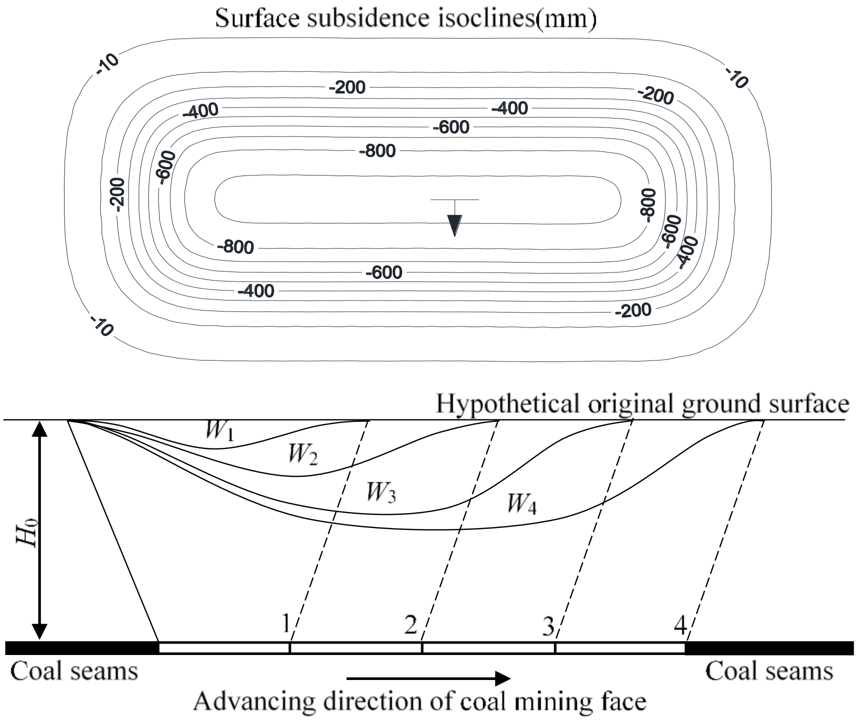

Figure S1. Diagram showing formation of ground surface subsidence caused by coal mining and surface subsidence isoclines to the face 4. Numbers 1, 2, 3 and 4 represent the locations of working faces. W1, W2, W3 and W4 are the subsidence basins corresponding to the working faces. H_0 is the depth to the coal seams.

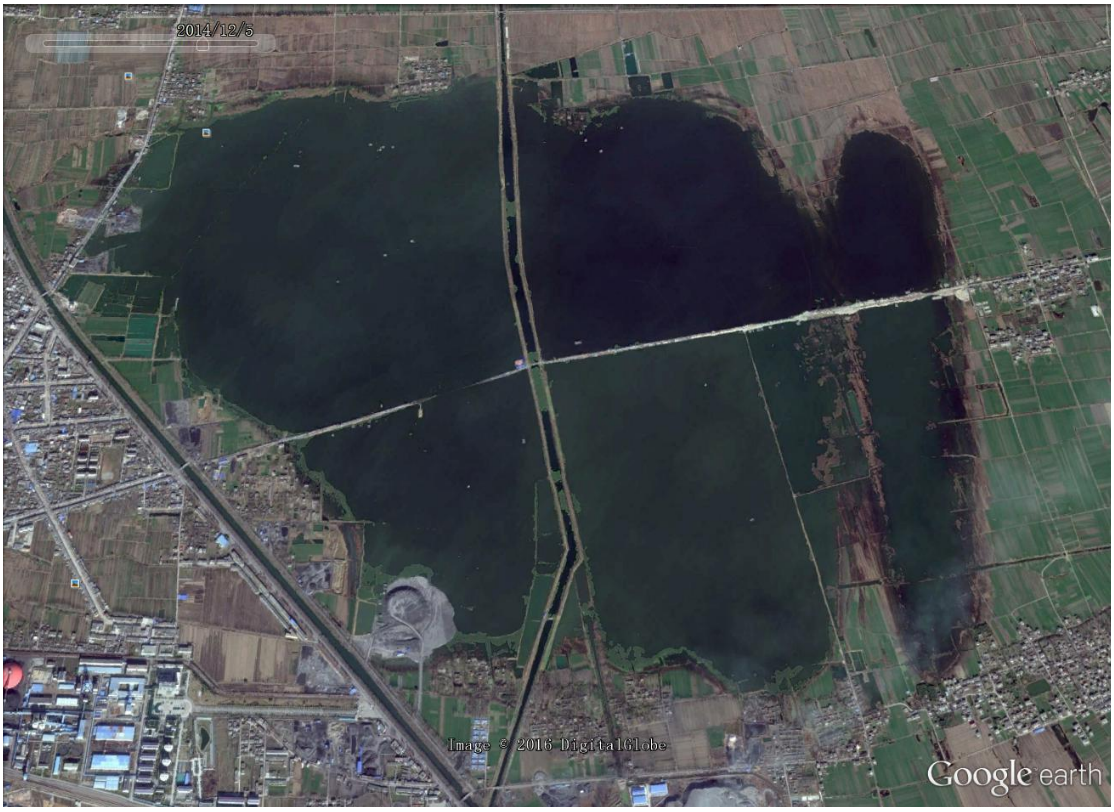

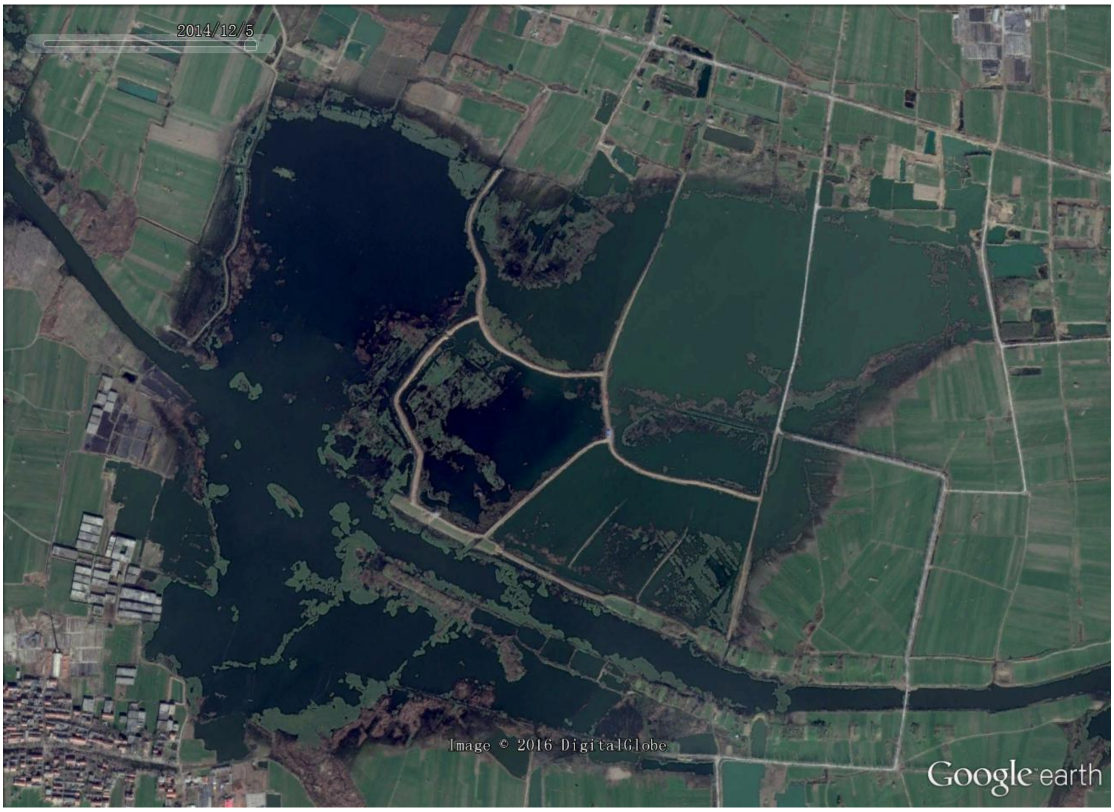

Figure S2. Landscape affected by coal mining land subsidence around the HCMSs. Images from Google earth.

The HCMSs, which include 7 sub-subsidence areas distributing independently from west to east, are passed through several tributaries of the Huaihe River including the Xifeihe, Yongxinghe, Jiahe and Niheihe rivers (Fig. S3). The hydraulic connection between these four rivers and the Yinghe and Cihuaixin Rivers has been cut off for flood controlling. These four basins are completely contained in the enclosed area surrounded by the Yinghe, Cihuaixin and Huaihe Rivers. While considering the hydrologic model used in this study coupling the groundwater numerical simulation module, boundary conditions are needed for the numerical simulation of groundwater. Yinghe, Cihuaixin and Huaihe Rivers can be used as head boundary as they are perennial rivers. In sum, it is reasonable and feasible to take the area surrounded by Yinghe, Cihuaixin and Huaihe Rivers as the study area (Fig. S3).

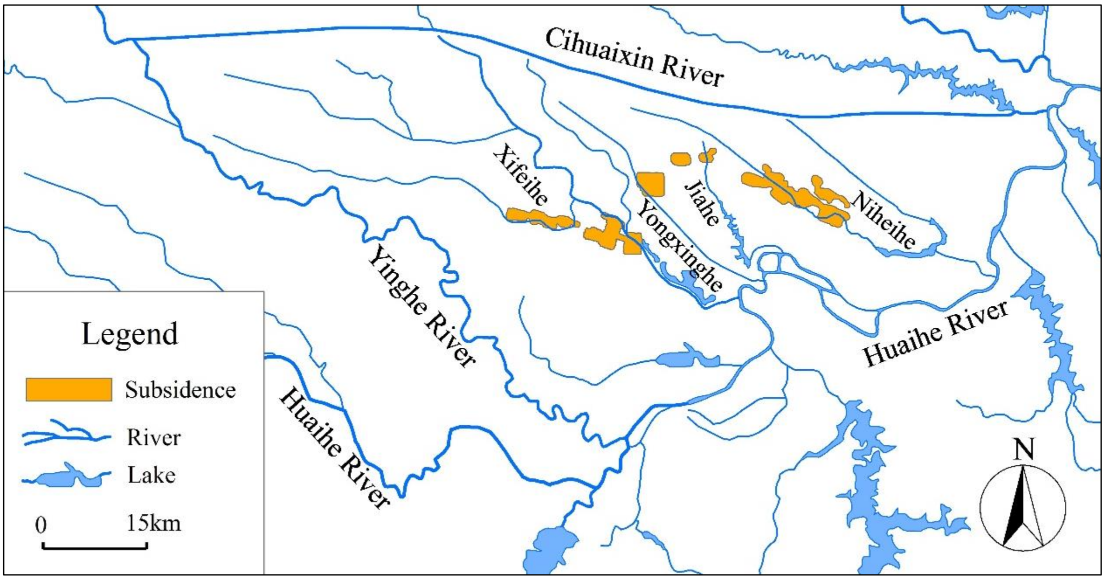

Figure S3. River system in the study area. Map was created using ArcGIS 9.3 (http://www.esri.com/software/arcgis/arcgis-for-desktop).

The location of the HRB in Greater China is shown in Fig. S4, and the location of the study area in the HRB is shown in Fig. S5. The study area, covering an area of 4,012 km^2, is located in the transitional zone between north and south climate zones of China; and is controlled by warm and semi-humid climate. The monsoon prevails in this region: winds blowing from the continent to the ocean in cold and dry winter, and from the ocean to the continent in summer warm and humid. The average temperature is about 15.8℃. The annual precipitation (weighted by the control area of the rainfall station) is 438–1,618 mm during 1954 to 2010, with an average of 888mm. The annual average pan evaporation (measured by E601 evaporator) from 1954 to 2010 is about 976 mm. Groundwater table is shallow in the study area with fluctuations around 1.5m. The coal mining and related industry and crop planting industries are the main economic revenues for this region.

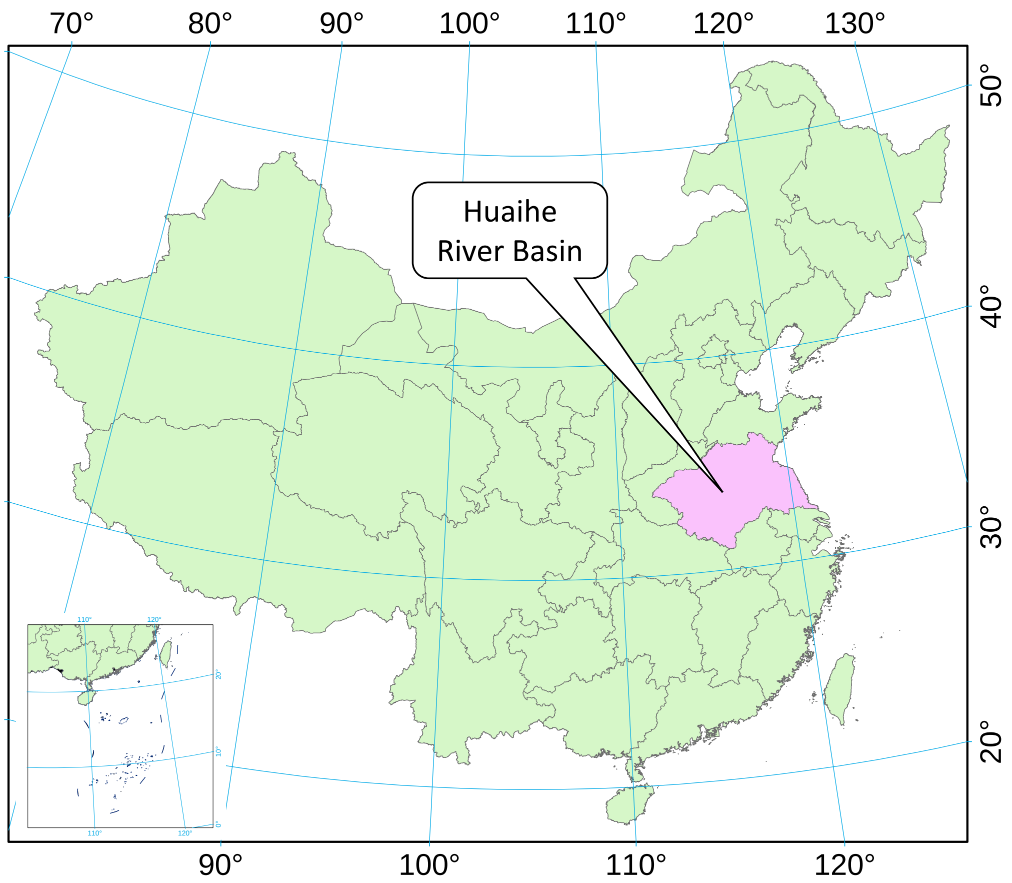

Figure S4. Location of the HRB in Greater China. Map was created using ArcGIS 9.3 (http://www.esri.com/software/arcgis/arcgis-for-desktop).

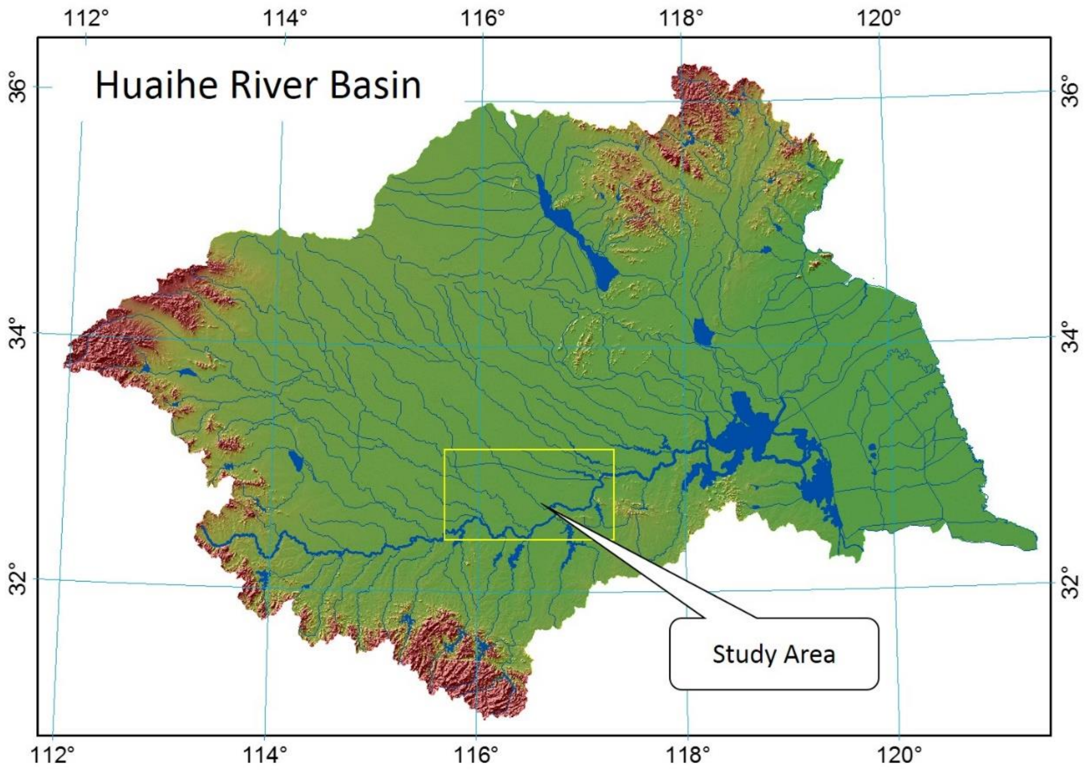

Figure S5. Location of the study area in the HRB. Map was created using ArcGIS 9.3 (http://www.esri.com/software/arcgis/arcgis-for-desktop).

Aquifers in the study area are Quaternary formations with the total thickness decreasing progressively from >500 m in the northwest to <100 m in the north and southeast. The Quaternary sediments consist of the Lower, Middle and Upper Pleistocene series, and the Holocene deposits. The boundary between the Lower and Middle Pleistocene series was not formally defined in this region. The thicknesses of the Lower and Middle Pleistocene (Q_{1-2}), Upper Pleistocene (Q_3) and Holocene (Q_4) sediments are 30–400m, 40–100m and 25–45m respectively. There is a less permeable layer composed of loam and clay on the top of Q_3 formation, and its thickness is about 10–35m. Other aquifers consist of fine sand, coarse sand, and silty sand, and contain several discontinuous weak permeable layers.

The landscape in this region is flat with a topographic slope of 1/15000-1/8000. The groundwater flow direction controlled by the topography is generally along the surface water flow. The small hydraulic gradient (approximately 1/10000) indicates that groundwater flow is slow and the lateral recharge is possibly limited.

Supplementary Information 2: Process scheme and conceptual model of MODCYCLE and its module "river-subsidence-groundwater" simulation module (RSGM)

2.1 The Modularized Model for Basin Scale Hydrologic Cycle (MODCYCLE)

The general model structure and hydrologic cycle simulation principle of the MODCYCLE code have been introduced in numerous Chinese monographs, journal papers and patents[2-4], but not yet reported in international journals. Here we present the framework and part principle of the model, and hope to help readers to understand the role of the model in the hydrologic cycle simulation of the subsidence.

2.1.1 Model structure and hydrologic cycle path in the MODCYCLE

The MODCYCLE is a distributed hydrologic simulation model based on physical mechanisms. In the planar structure, the model firstly divides the river basin into several subbasins using the digital elevation model (DEM). Relationship among subbasins is built by the topology of main channels, which are generated following the corresponding subbasins. Then each subbasin is further divided into a plurality of basic

simulation units (BSUs) according to the space distribution of land use & land cover (LUC), soil and land management operation (MGT). The BSU in fact is an aggregate that has the same LUC, soil and MGT. The BSUs may be scattered in the subbasin, and are not connected each other. In addition to the BSUs, the subbasins can include open waters such as swamps, wetlands, ponds, and lakes. The aquifers below the vadose zone are divided into two layers, the shallow layer and the deep layer, in each subbasin. To simplify the vertical flow simulation, the model only simulates the interaction between the shallow and deep groundwater system. The groundwater in subbasin aquifer can be independent or interconnected depending on the specific hydraulic conditions. Figures S6 and S7 show the diagram of planar structure and the hydrologic cycle process of the MODCYCLE, respectively.

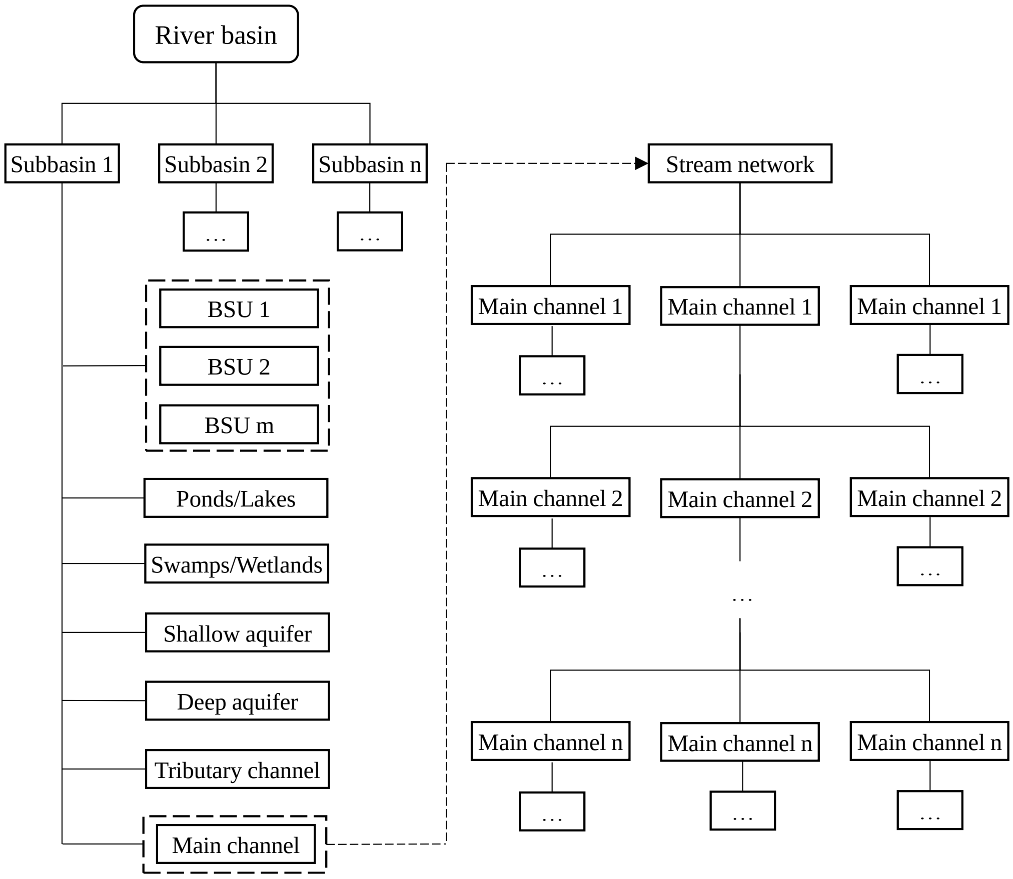

Figure S6. Model structure of the MODCYCLE (translated from Lu et al., 2012).

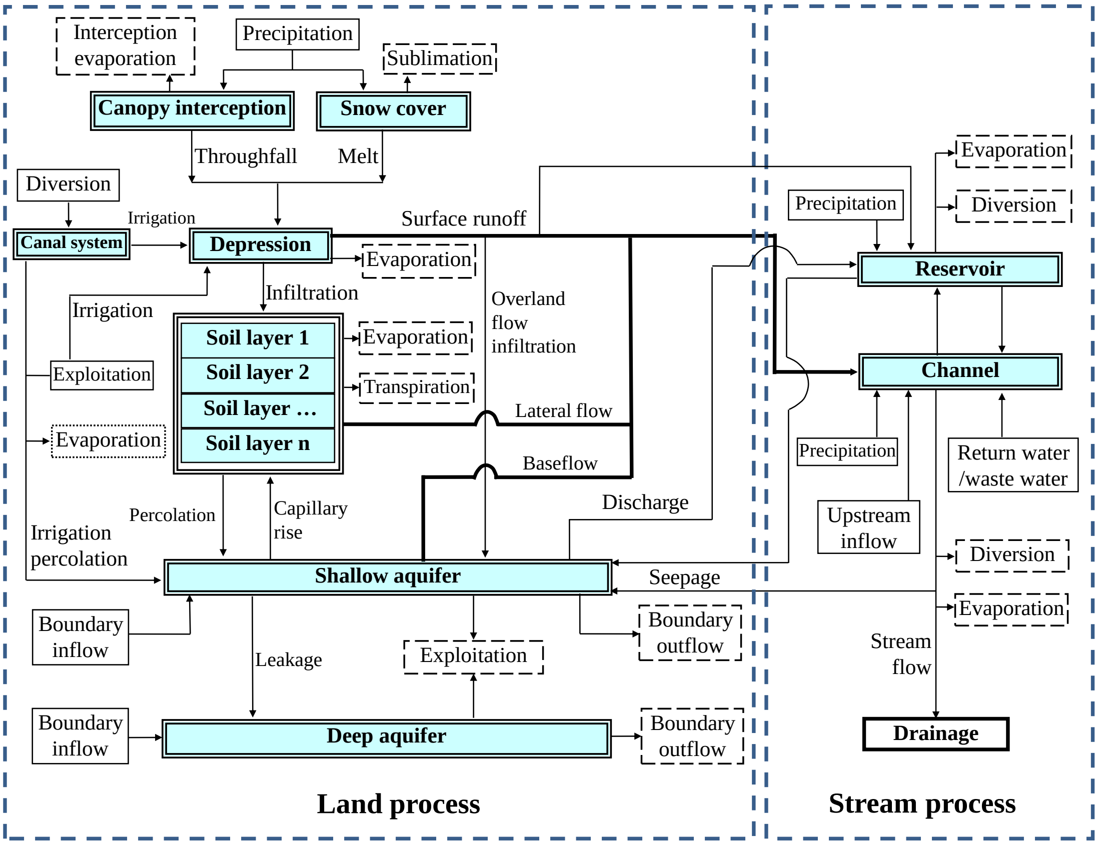

Figure S7. Hydrologic cycle path of the MODCYCLE (translated from Lu et al., 2012).

2.1.2 Simulating principle of surface water – groundwater interaction

In the current version of the MODCYCLE, subbasins can be divided into mountain area subbasins and plain area subbasins to simulate the groundwater respectively. Subbasins in mountain areas generally have natural watersheds, which are often corresponding for surface runoff and groundwater flow in the same subbasin. Therefore, equilibrium mode is used in the groundwater simulation of mountain area subbasins, in which groundwater in each subbasin is independent and do not consider the groundwater lateral exchange among different subbasins. Dividing crests of subbasins in plain areas are not obvious, groundwater in subbasins are inter-connective, and the lateral flow of groundwater is simulated explicitly.

In the MODCYCLE, the interaction between surface water and groundwater in plain areas is based on coupling the distributed hydrologic model and groundwater flow simulation component, and achieved by real-time information interaction between the two models. The groundwater flow simulation model is based the three dimensional

groundwater flow equation, which is solved using the block-centered finite difference method. The governing equation is same with the MODFLOW code[5]:

$$\frac{\partial}{\partial x}\left(K_{xx}\cdot\frac{\partial h}{\partial x}\right)+\frac{\partial}{\partial y}\left(K_{yy}\cdot\frac{\partial h}{\partial y}\right)+\frac{\partial}{\partial z}\left(K_{zz}\cdot\frac{\partial h}{\partial z}\right)-W=S_s\cdot\frac{\partial h}{\partial t} \quad \text{(S1)}$$

where K_{xx}, K_{yy}, and K_{zz} are the components of hydraulic conductivity along coordinate *X*, *Y*, and *Z*, which are assumed to be parallel to the major axes of hydraulic conductivity (LT^{-1}) , *h* is the potentiometric head (L), *W* is a volumetric flux per unit volume representing sources and/or sinks of water (T^{-1}), S_S is the specific storage of the porous material (L^{-1}) , and *t* is time (T).

Spatial superposition between subbasins and groundwater numerical simulation grid cells

The superposition of subbasins and grid cells is diagramed in Fig. S8. Cutting the grid cells using the boundary line of subbasins, the area proportions of grid cells (including whole and partial cells) in the corresponding subbasin can be calculated as:

$$PA_{iCell,\,jSubb}=A_{iCell,\,jSubb}/A_{jSubb} \quad \text{(S2)}$$

where $PA_{iCell,\,jSubb}$ is the area proportion of the part of grid cell with number *iCell* inside the subbasin with number *jSubb* to the whole area of the subbasin, $A_{iCell,\,jSubb}$ is the overlap area of the grid cell with number *iCell* and the subbasin with number *jSubb* (L^2), and A_{jSubb} is the area of the subbasin with number *jSubb* (L^2).

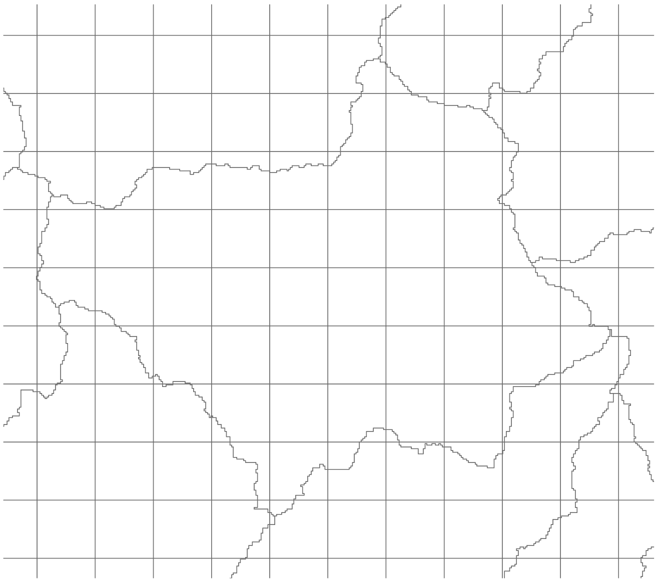

Figure S8. Superposition of hydrologic model subbasins and groundwater numerical simulation grid cells. The bend lines are the boundary of subbasins, and the straight lines constitute grid. Map was created using ArcGIS 9.3 (http://www.esri.com/software/arcgis/arcgis-for-desktop).

By using the above method, the spatial correlation between subbasins for hydrologic model and grid cells for groundwater flow simulation is established, which provides the basis for the two-way information exchange between the two models.

Information exchange between hydrologic model and groundwater flow model

Vertical fluxes calculated by the hydrologic model are transferred to the groundwater flow model, in which these fluxes are treated as sources or sinks. Spatial and temporal distribution of vertical fluxes include precipitation percolation, surface water (river, reservoir, wetland, and canal so on) seepage, groundwater irrigation return flow, baseflow, phreatic evaporation, and groundwater withdrawal. Groundwater flow model calculates the groundwater head and depth using the above fluxes, and then transfer these level and depth to the hydrologic model for hydrologic simulation. Flow chart of information exchange between the two systems is shown in Fig. S9.

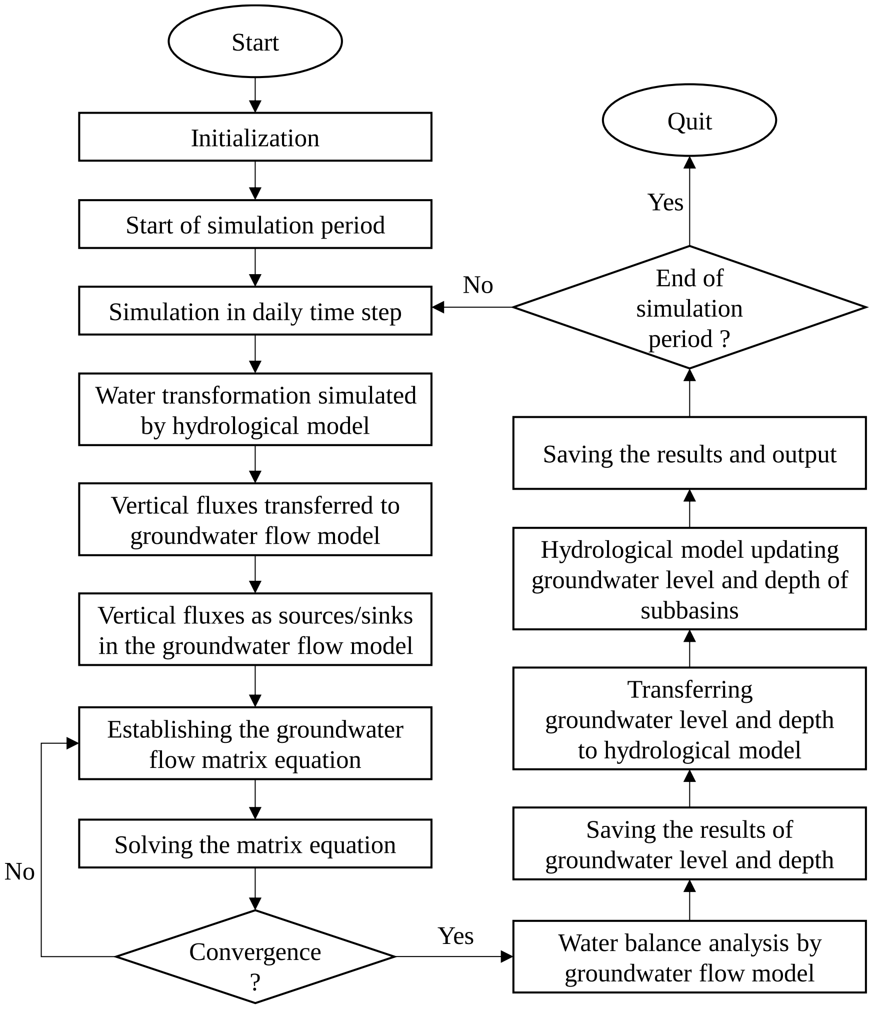

Figure S9. Flow chart of synchronous coupling between hydrologic simulation system and groundwater flow simulation system.

In a daily time step, the above "synchronous coupling" includes the following sub steps:

Step 1: The hydrologic model implements hydrologic cycle simulation in the time step firstly, and calculate every vertical flux of groundwater in each subbasin:

$$\begin{aligned} P_{jSubb} &= p_r + p_w + p_i \\ D_{jSubb} &= d_e + d_b + d_p \end{aligned} \tag{S3}$$

where P_{jSubb} is the sum of vertical recharge to groundwater in the subbasin with number *jSubb*(L^3T^{-1}), p_r, p_w, and p_i are precipitation percolation, surface water (river, reservoir, wetland, and canal so on) seepage, and well irrigation return to groundwater respectively(L^3T^{-1}); D_{jSubb} is the sum of vertical discharge from groundwater in the subbasin with number *jSubb*(L^3T^{-1}), and d_e, d_b, and d_p are phreatic evaporation, baseflow, and groundwater withdrawal respectively(L^3T^{-1}).

Step 2: The vertical fluxes of each subbasin simulated by the hydrologic model are transferred to the groundwater numerical model, which is based on the spatial relation between the subbasins and the grid cells and the area proportion of grid cell to subbasin. Water sources and sinks of each grid cell can be calculated by:

$$W_{iCell} = \sum_{jSubb}^{Sum} (P_{jSubb} - D_{jSubb}) \cdot PA_{iCell,\, jSubb} \tag{S4}$$

where W_{iCell} is the total net water source (+) or sink (-) of the grid cell with number *iCell*(L^3T^{-1}), *sum* is the total number of subbasins in the whole simulation space, and the meaning of other symbols can be found in the above.

Step 3: After the above steps, the groundwater flow model has got the sources or sinks in the time step (*W* in equation S1). Groundwater head and depth of all grid cells are updated after converge of the groundwater flow simulation.

Step 4: The groundwater tables in grid cells are transferred to the hydrologic model. The average groundwater heads and depths in subbasins will be updated by the area weighted average method, which also based on the spatial relation between the subbasins and the grid cells and the area proportion of grid cell to subbasin.

$$H_{jSubb} = \sum_{iCell}^{Total} h_{iCell} \cdot PA_{iCell,\, jSubb}$$
$$D_{jSubb} = \sum_{iCell}^{Total} d_{iCell} \cdot PA_{iCell,\, jSubb} \quad \text{(S5)}$$

where H_{jSubb} and D_{jSubb} are average groundwater head and depth of the subbasin with number *jSubb*(L), h_{iCell} and d_{iCell} are groundwater head and depth of the grid cell with number *iCell* from **step 3**, *Total* is the total number of grid cells in the whole simulation space, and the meaning of other symbols can be found in the above.

Step 5: After the above steps, the simulation of the current time step is finished. The coupling model system will conduct the simulation of the next time step. Firstly, the hydrologic model will simulate the hydrologic cycle using the groundwater level and depth of subbasins updated in last time step. The above steps are repeated until all the time steps are completed, then end the whole simulation period.

The MODCYCLE combines the hydrologic process and groundwater flow as a complete system, in which groundwater simulation component of the model can obtain sources and sinks information in real time from the hydrologic model. The groundwater simulation of MODCYCLE is similar to MODFLOW. However, MODFLOW needs the sources/sinks to be processed artificially, and simulates groundwater independently.

The coupling principle of MODCYCLE is based on spatial integration between subbasins of hydrologic model and grid cells of groundwater model. The SHE code in DHI Mike software uses the complete grid from top to bottom in the coupled simulation of surface water and groundwater, and the grid size in all profiles are the same in horizontal direction. It will increase the amount of data and reduce the model running efficiency in order to maintain the accuracy of simulation in large basins or regions. The MODCYCLE can partition subbasins and grid cells flexibly according to the different conditions of landscape and aquifer, reduce unnecessary data redundancy and therefore can be applied to large basins or regions. In addition, the MODCYCLE can be divided into hilly areas and plain areas, and conduct water balance calculations or numerical simulation respectively. Hydraulic information is generally limited for aquifers in hill areas, therefore, it is more appropriate to use water balance calculations, which can reduce the amount of computation.

2.2 RSGM module of the MODCYCLE for the simulation of hydrologic cycle in coal mining subsidence areas

Governing equation, spatial discretization, and simulation principle of the conversion relationship between the subsidence area water and the groundwater for the RSGM have been introduced in the main text. The calculation principle of the recharge and discharge for the subsidence areas are presented in this report.

2.2.1 Precipitation and evaporation on the water areas

Precipitation P (L^3T^{-1}) and evaporation E (L^3T^{-1}) on the water areas are calculated using meteorological data and water area:

$$\begin{cases} P = \bar{A}_w \cdot P_{day} \\ E = \bar{A}_w \cdot ET_{day} \end{cases} \tag{S6}$$

where P_{day} and ET_{day} are the precipitation on the water surface and the evaporation from the water surface for a daily time step(LT^{-1}), and $\bar{A}_w$ is the water area(L^2).

2.2.2 Flow from upstream

Flow from upstream Q_{up} is the total water of all rivers into the subsidence area which derived from the hydrologic simulation of the MODCYCLE:

$$Q_{up} = \sum Q_{up}^i \tag{S7}$$

where Q_{up}^i is the flow into the subsidence from the i-th upstream river (L^3T^{-1}).

2.2.3 No-water area runoff

No-water area runoff R_{nw} (L^3T^{-1}) is calculated by the modified SCS curve number equation[6]:

$$\begin{cases} R_{nw} = \dfrac{(P_{day} \text{-} 0.2S)^2}{(P_{day} + 0.8S)} \times \bar{A}_{nw} \\ S = 25.4(\dfrac{1000}{CN} - 10) \end{cases} \tag{S8}$$

where S is the retention parameter, which varies spatially due to changes in soils, land use, management and slope and temporally due to changes in soil water content(LT^{-1}), CN is the curve number for a daily time step, $\bar{A}_{nw}$ is the no-water area(L^2), and the meaning of other symbol can be found above.

2.2.4 Water withdrawal

Water withdrawal W (L^3T^{-1}) from the subsidence area is use for irrigation, industry, livelihood, and ecology:

$$W = w_{irri} + w_{indu} + w_{live} + w_{eco} \tag{S9}$$

where w_{irri}, w_{indu}, w_{live}, and w_{eco} are water withdrawal from the subsidence area for irrigation, industry, livelihood, and ecology, respectively.

2.2.5 Drainage through outlet

Drainage trough the outlet of the subsidence Q_{dr} (L^3T^{-1}) is calculated by the modified flow equation of the broad-crested weir[7]:

$$Q_{dr} = mB_0\sqrt{2g}H_0^{3/2} \tag{S10}$$

where m is the dimensionless discharge coefficient (-), B_0 is the channel breadth (L), g is the gravity acceleration (LT^{-2}), and H_0 is the upstream total head above the crest (L).

2.2.6 Groundwater discharges from aquifer under the water area

The calculations of recharge and discharge of groundwater in the subsidence area are similar to MODFLOW in simulations of river, reservoir and lake. Groundwater discharges from aquifers into the subsidence area water when the groundwater level is higher than the water level in subsidence area (Fig. S10).

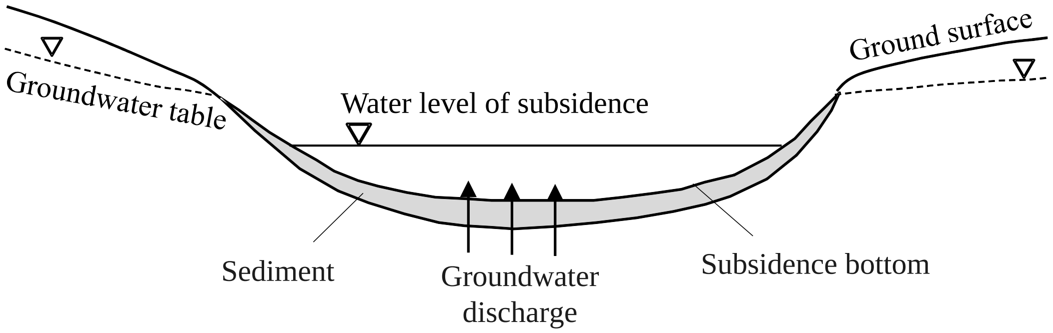

Figure S10. Groundwater discharges into the subsidence area water under the water area.

The total groundwater discharge from aquifer to the subsidence area water is the sum of groundwater discharge from all grid cells under the water area, calculated by equation (S11):

$$G_{gw}^{in} = \sum_{k=1}^{K} q_k^{in} = \sum_{k=1}^{K} C_{s,k}(h_{a,k} - \overline{h}_l) \quad when \quad h_{a,k} > \overline{h}_l \tag{S11}$$

where G_{gw}^{in} is the discharge into subsidence area from aquifer under the water area (L^3T^{-1}), K is the total number of the grid cells discharging groundwater under the water

area, q_k^{in} is the discharge from the k-th grid cell (L^3T^{-1}), $C_{s,k}$ is the transmissivity between the subsidence bottom and aquifer for the k-th grid cell (L^2T^{-1}), $h_{a,k}$ is the groundwater level in the k-th grid cell under the water area (L), and $\bar{h}_l$ is the average water level in the subsidence area (L).

2.2.7 Groundwater discharge from aquifer under the no-water area

When the groundwater table is higher than the water level in the subsidence area, there is a no-water area between the two water levels, where groundwater will discharge into subsidence area (Fig. S11).

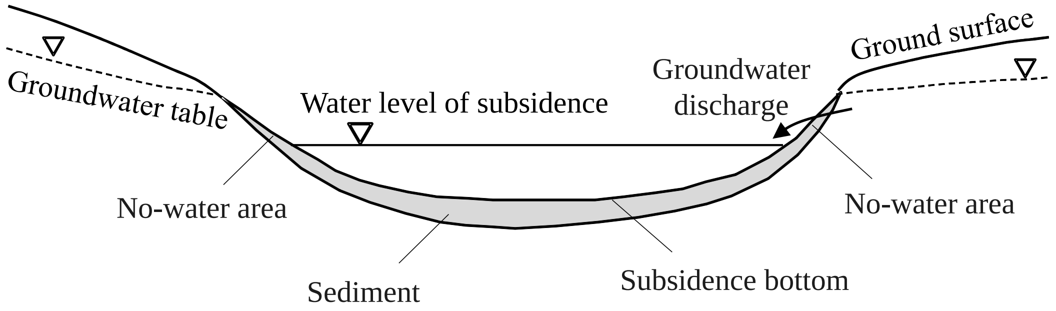

Figure S11. Groundwater discharges into the subsidence area under the no-water area.

Groundwater discharge under no-water area is calculated according to the difference between the groundwater level and the bottom elevation of the grid cell:

$$G_{nw}^{in} = \sum_{m=1}^{M} q_m^{in} = \sum_{m=1}^{M} C_{s,m}\left(h_{a,m} - Btm_m\right) \quad when \quad h_{a,m} > Btm_m \tag{S12}$$

where G_{nw}^{in} is the recharge into subsidence from aquifer under the no-water area(L^3T^{-1}), M is the total number of the grid cells discharging groundwater under the no-water area, q_m^{in} is the discharge from the m-th grid cell (L^3T^{-1}), $C_{s,m}$ is the transmissivity between the subsidence bottom and aquifer for the m-th grid cell (L^2T^{-1}), $h_{a,m}$ is the groundwater head of the m-th grid cell under the no-water area (L), and Btm_m is the average elevation of the subsidence bottom on the m-th grid cell (L).

2.2.8 Leakage into aquifer

Leakage occurs when the groundwater level in grid cells is lower than the average water level of the subsidence. There are two forms of leakage according to the relative position between the bottom elevation and the groundwater table of the grid cell: leakage associated with the groundwater table and leakage not associated with the

groundwater table (or independent leakage) (Fig. S12).

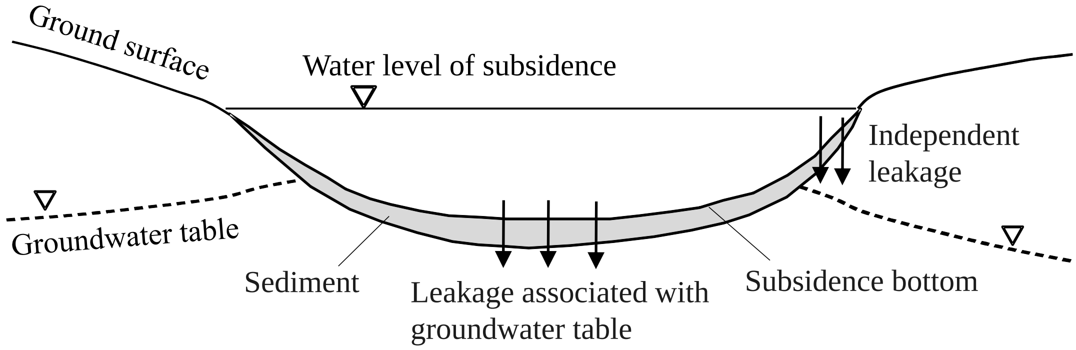

Figure S12. Leakage of subsidence area water into groundwater.

Leakage of subsidence G_{gw}^{out} is calculated as:

$$\begin{cases} G_{gw}^{out,1} = \sum_{n=1}^{N} q_n^{out} = \sum_{n=1}^{N} C_{s,n}(\overline{h_l} - h_{a,n}) \quad when \quad \overline{h_l} > h_{a,n} > Btm_n \\ G_{gw}^{out,2} = \sum_{s=1}^{S} q_s^{out} = \sum_{s=1}^{S} C_{s,s}(\overline{h_l} - Btm_s) \quad when \quad \overline{h_l} > Btm_s > h_{a,s} \\ G_{gw}^{out} = G_{gw}^{out,1} + G_{gw}^{out,2} \end{cases} \tag{S13}$$

where $G_{gw}^{out,1}$ is the leakage associated with groundwater table (L^3T^{-1}), N is the total number of grid cells where groundwater tables are higher than their the bottoms, q_n^{out} is the leakage of the n-th grid cell (L^3T^{-1}), $C_{s,n}$ is the transmissivity between the subsidence bottom and aquifer for the n-th grid cell (L^2T^{-1}), $h_{a,n}$ is the groundwater head of the n-th grid cell(L), Btm_n is the average elevation of the subsidence bottom on the n-th grid cell (L), $G_{gw}^{out,2}$ is the leakage not associated with groundwater table (L^3T^{-1}), S is the total number of grid cells whose groundwater tables are lower than their the bottoms, q_s^{out} is the leakage of the s-th grid cell (L^3T^{-1}), $C_{s,s}$ is the transmissivity between the subsidence bottom and aquifer for the s-th grid cell (L^2T^{-1}), $h_{a,s}$ is the groundwater head of the s-th grid cell (L), Btm_s is the average elevation of the subsidence bottom on the s-th grid cell (L), and the meaning of other symbol can be found in the above.

Figure S13 shows the flow chart of program design for the “river-subsidence-groundwater” simulation module (RSGM). The water budget items of a subsidence area

associated with groundwater table need to be iteratively calculated in RSGM, so it should be coupled in the groundwater flow model. The flow chart only presents the calculation part of the module, ignores the input and output and their processing procedures, and describes the calculation for a single subsidence area.

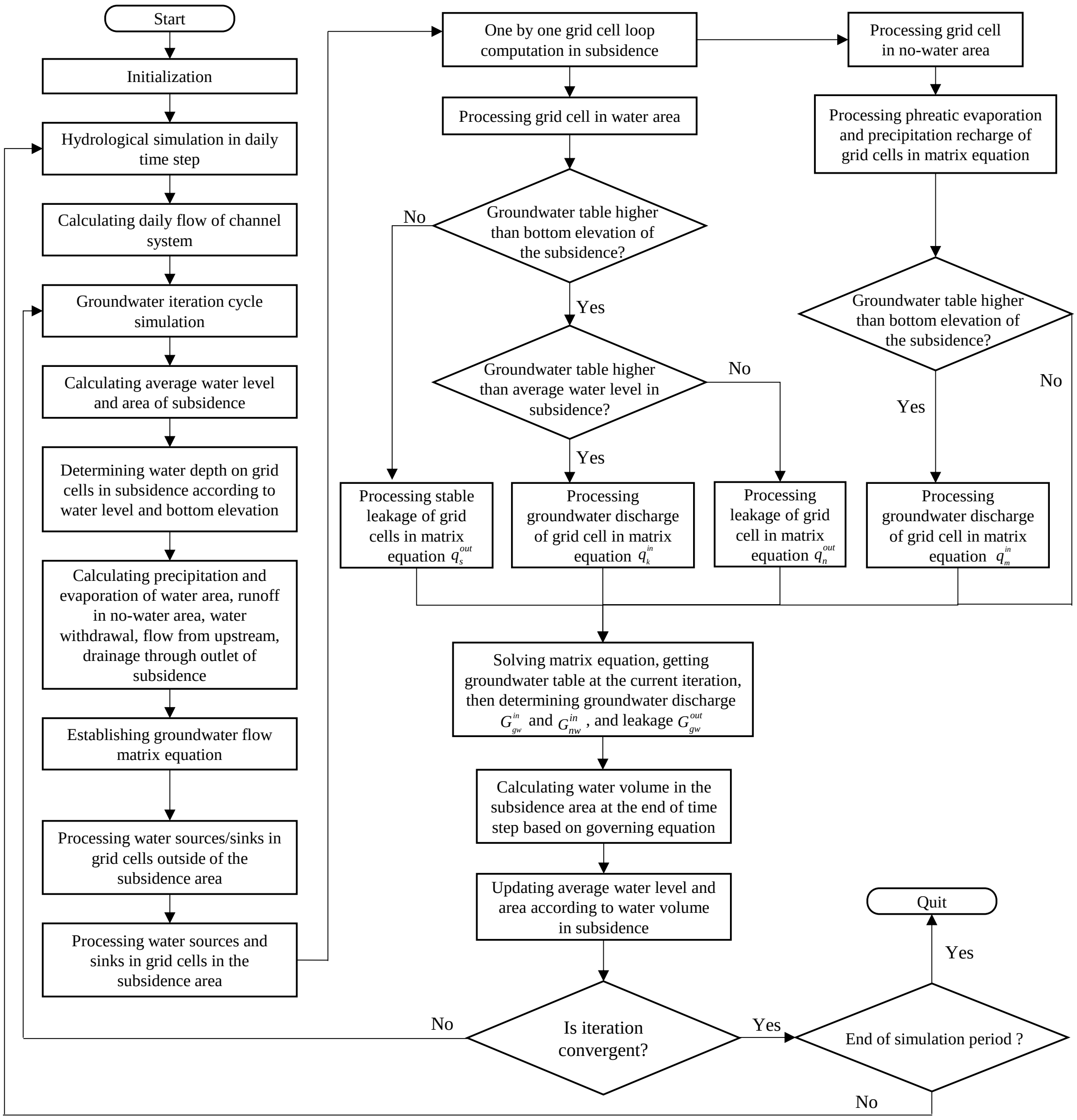

Figure S13. Flow chart of program design for the RSGM module of the MODCYCLE.

Supplementary Information 3: Data and its processing for model building

3.1 Main spatial data and its processing

3.1.1 Subbasin and main channel

In this simulation study, the model domain was divided into 1245 subbasins, each of which has its own main channel. Subasins and their main channels are presented in Fig.

S14.

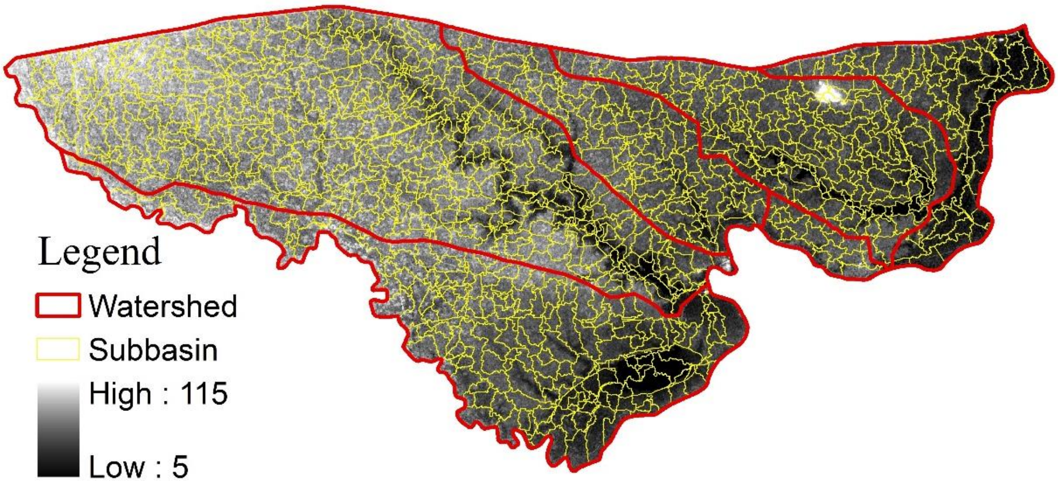

a. Subbasins

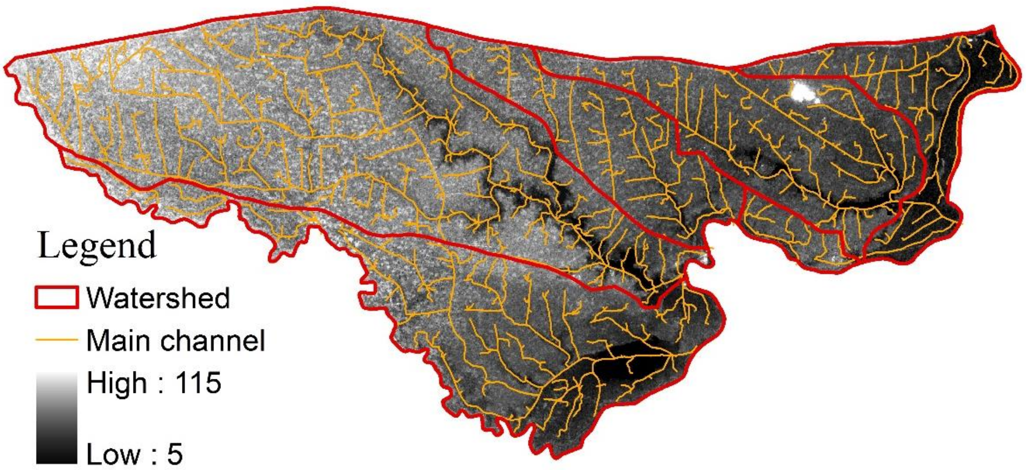

b. Main channels

Figure S14. (a) Subbasins and (b) main channels in the study area. Maps were created using ArcGIS 9.3 (http://www.esri.com/software/arcgis/arcgis-for-desktop).

3.1.2 Grid for groundwater flow simulation

In our study, the model grid was divided into two layers, representing the shallow and deep aquifers, respectively. Each model layer consists of 132 rows and 276 columns with a uniform size of 500m×500m. Each layer has 36432 cells in which 16166 cells are in the study area and others are inactive (Fig. S15).

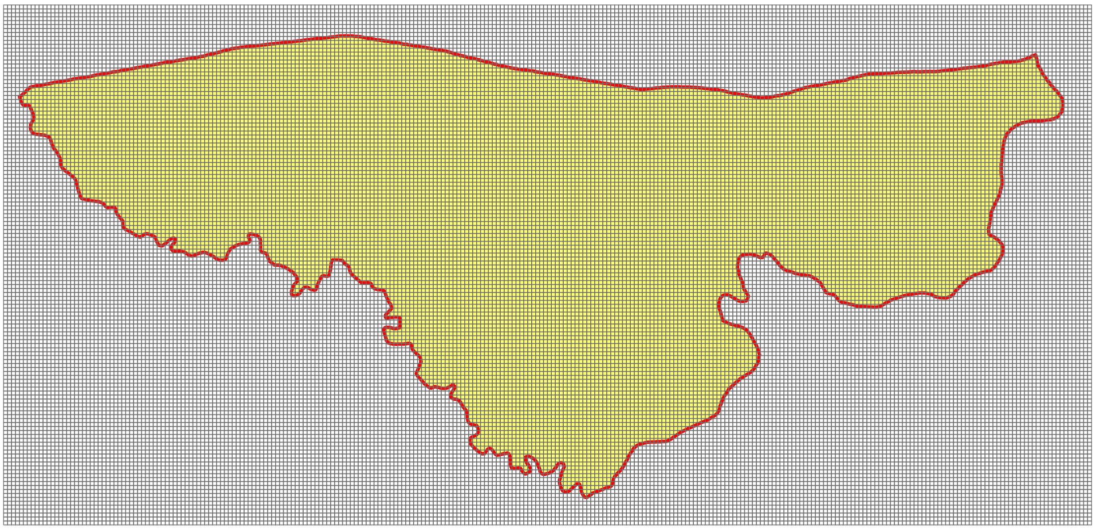

Figure S15. Grid used in the groundwater flow simulation. Map was created using ArcGIS 9.3 (http://www.esri.com/software/arcgis/arcgis-for-desktop).

3.1.3 Land use and cover

Land use and cover (LUC) types in the study area include agricultural land, forest land, grassland, water area, industrial and mining land, resident land, etc., in which the agricultural land has the largest area, accounting for approximately 80% of the total area, followed by the residential land (about 14%), and other types (less than 5%). Spatial distribution of the LUC types is shown in Fig. S16.

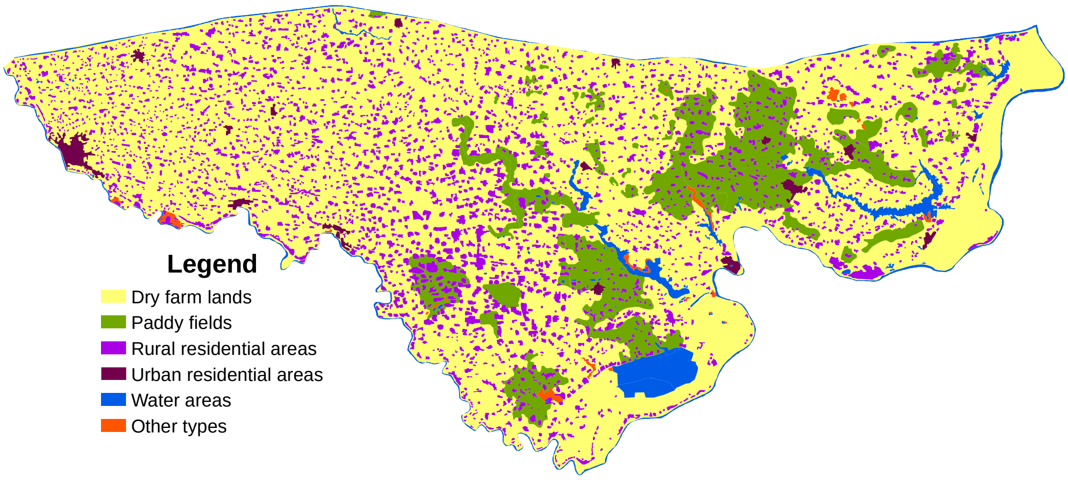

Figure S16. Land use and cover (LUC) types in the study area. Map was created using ArcGIS 9.3 (http://www.esri.com/software/arcgis/arcgis-for-desktop).

3.1.4 Soil types

Spatial distribution of soil types in study area is presented in Fig. S17. Physical

properties of those soil types can derived from the China Soil Scientific Database (http://www.soil.csdb.cn/).

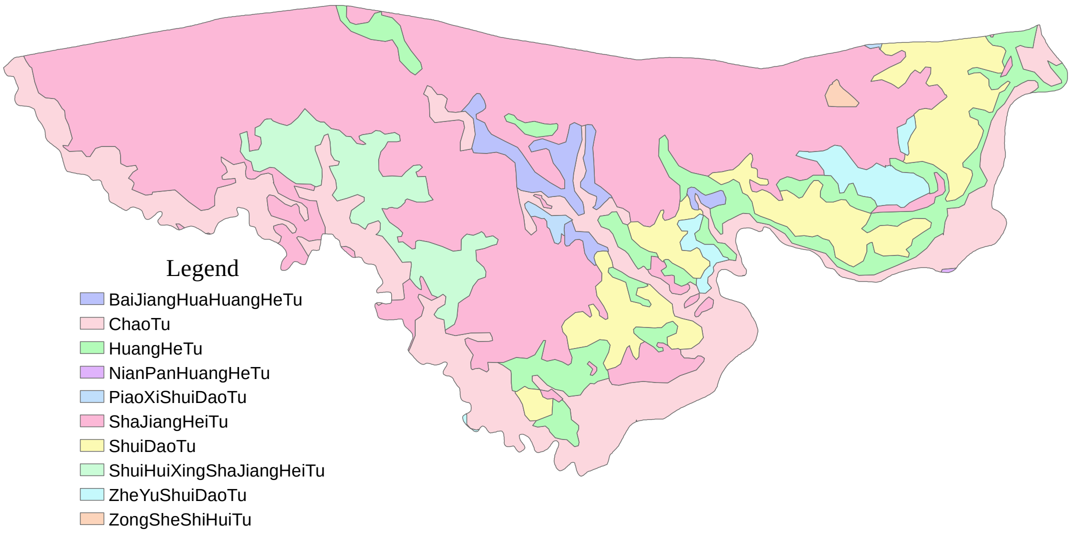

Figure S17. Soil types in the study area. Map was created using ArcGIS 9.3 (http://www.esri.com/software/arcgis/arcgis-for-desktop).

3.1.5 Weather stations in subbasins

Spatial distribution of weather data measured at stations is processed using a method similar to the Thiessen polygons. Weather information at the station in a subbasin or the nearest one is used as the weather input for the subbasin, as shown in Fig. S18 and Fig. S19.

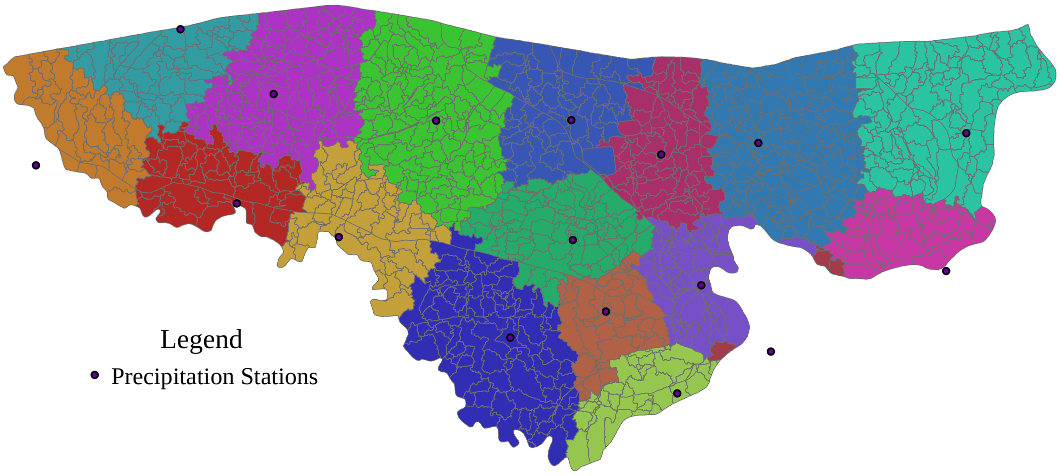

Figure S18. Subbasins and their corresponding precipitation stations. Map was created using ArcGIS 9.3 (http://www.esri.com/software/arcgis/arcgis-for-desktop).

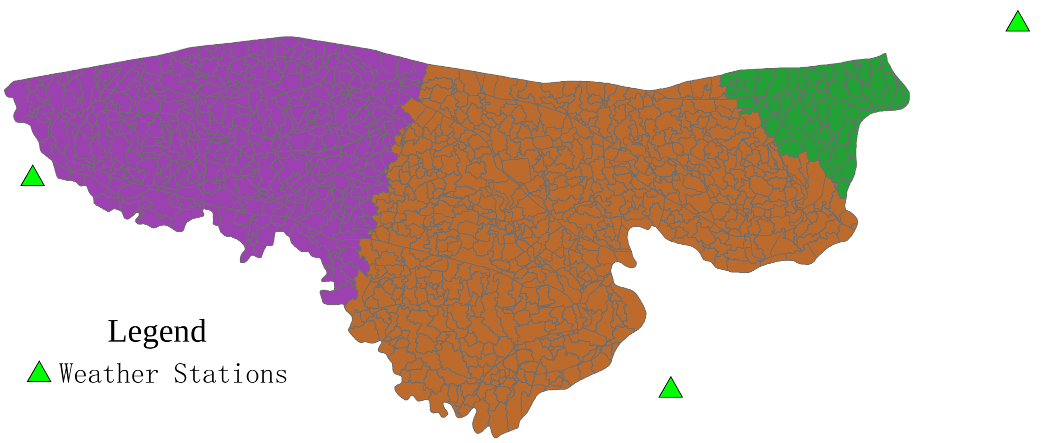

Figure S19. Weather stations with maximum/minimum air temperature, solar radiation, wind speed and relative humidity and their corresponding subbasins. Map was created using ArcGIS 9.3 (http://www.esri.com/software/arcgis/arcgis-for-desktop).

3.1.6 Coal mining subsidence data

Coal mining subsidence data was provided by the China University of Mining and Technology (CUMT). The data are presented in the form contour maps of subsidence depth (Fig. S20).

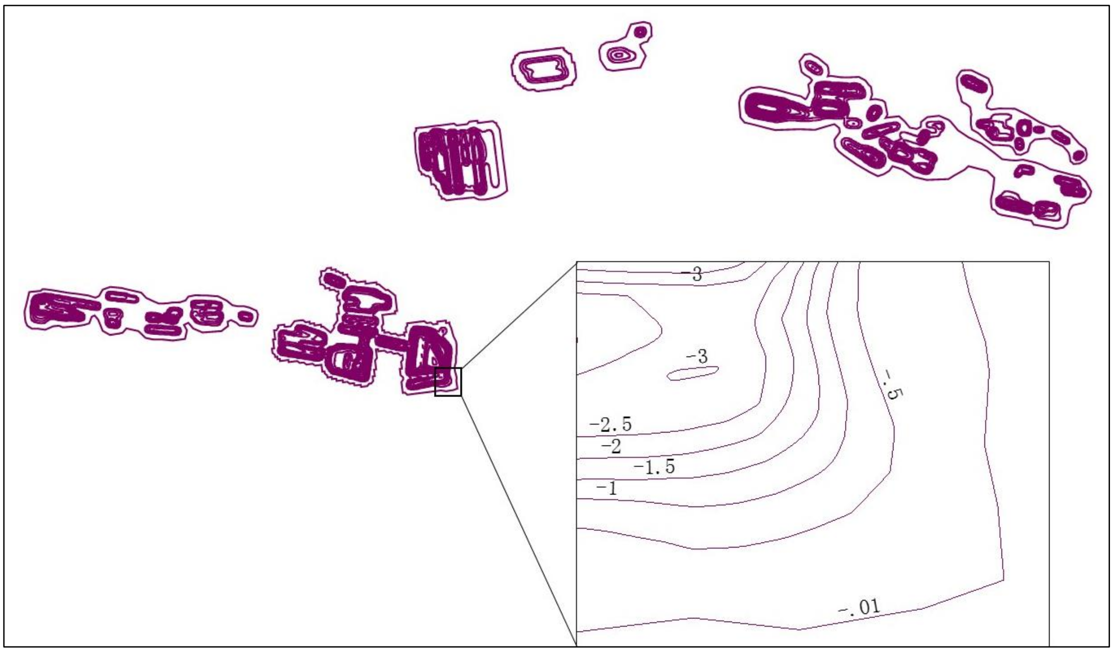

Figure S20. Contour map of the coal mining subsidence depth (in unit of meter). Map was created using ArcGIS 9.3 (http://www.esri.com/software/arcgis/arcgis-for-desktop).

3.1.7 Hydrogeological parameters

Main hydrogeological information and parameters used in the groundwater flow

simulation of the study area include ground surface elevation, specific yield, transmissivity and water aquifer bed elevation, etc. (Fig. S21).

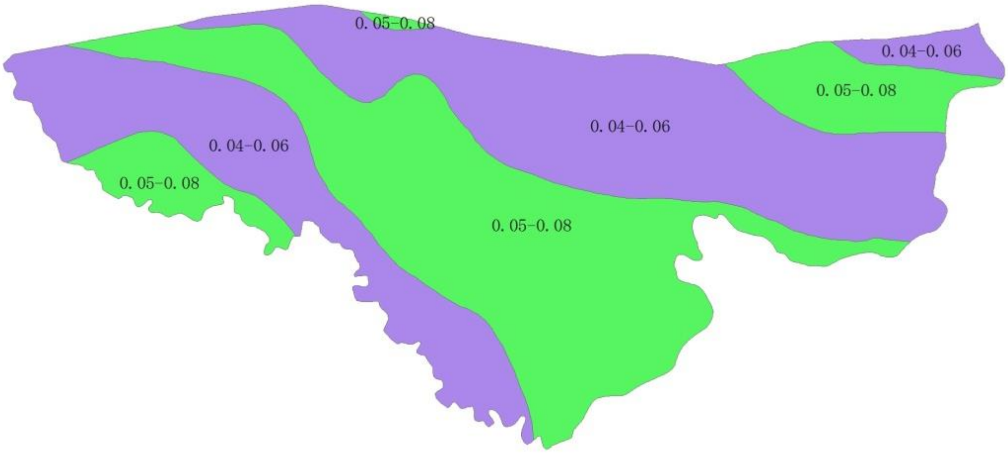

a. Specific yield of the shallow aquifer

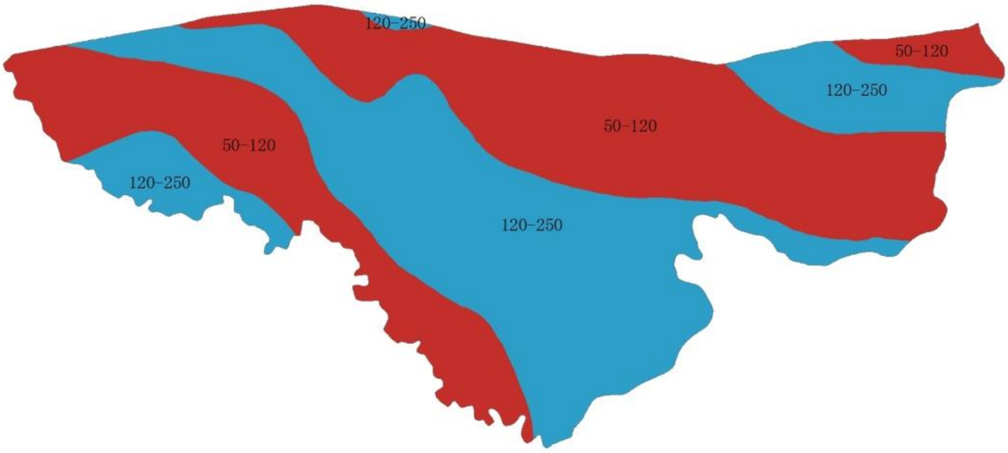

b. Transmissivity of the shallow aquifer (m^2/day)

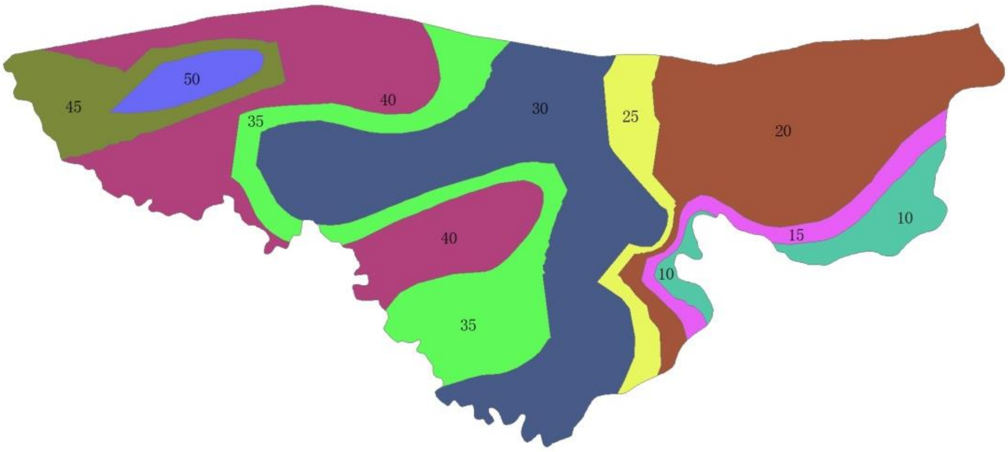

c. Depth to the bottom of shallow aquifer (m)

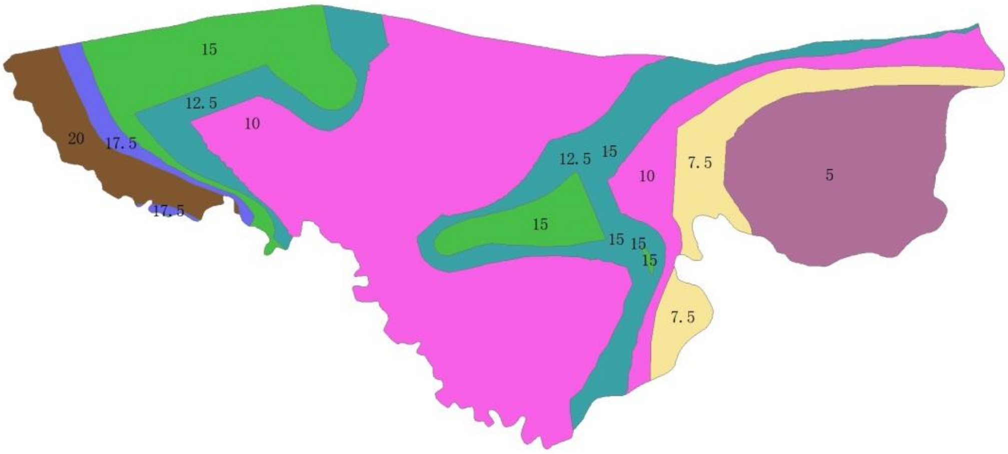

d. Thickness of the shallow aquifer (m)

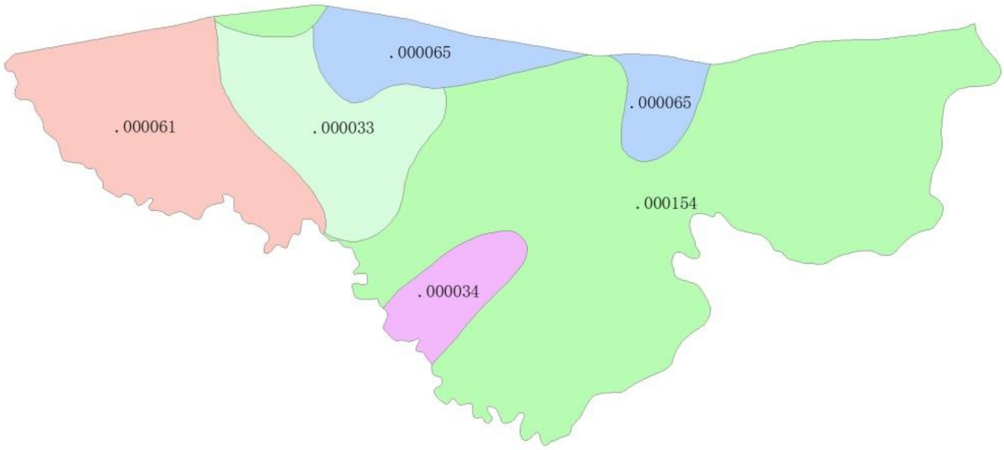

e. Vertical hydraulic conductivity divided by the thickness of the less permeable layer (s^{-1})

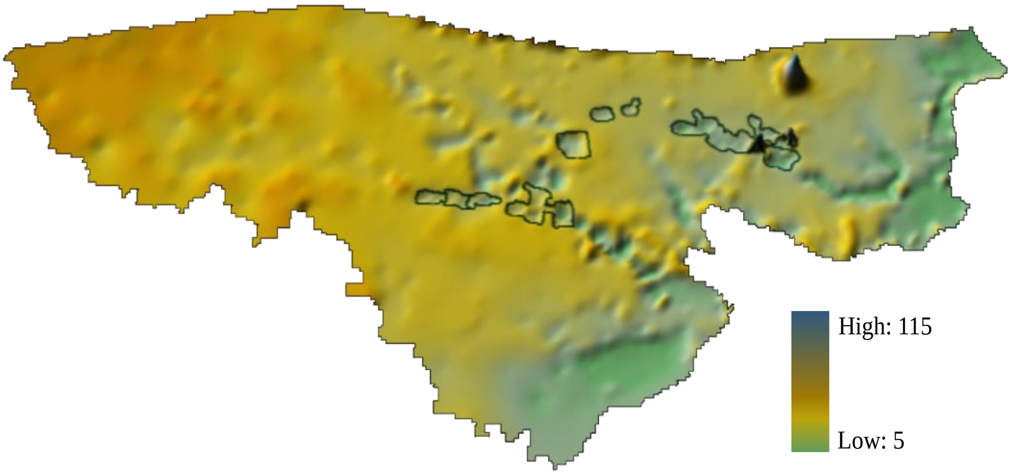

f. Ground surface elevation generated using DEM and measured elevation points (m). Map was created using Surfer 12.7 (http://www.goldensoftware.com/products/surfer).

Figure S21. Main hydrogeological parameters used in the groundwater flow simulation. Maps (a-e) were created using ArcGIS 9.3 (http://www.esri.com/software/arcgis/arcgis-for-desktop).

3.2 Main driving factors for hydrologic cycle

3.2.1 Weather information

Weather data used in the MODCYCLE, including daily precipitation, maximum/minimum air temperature, solar radiation, wind speed and relative humidity during 2001 to 2010, come from 17 precipitation stations and 3 national basic weather stations mentioned above (Fig. S22).

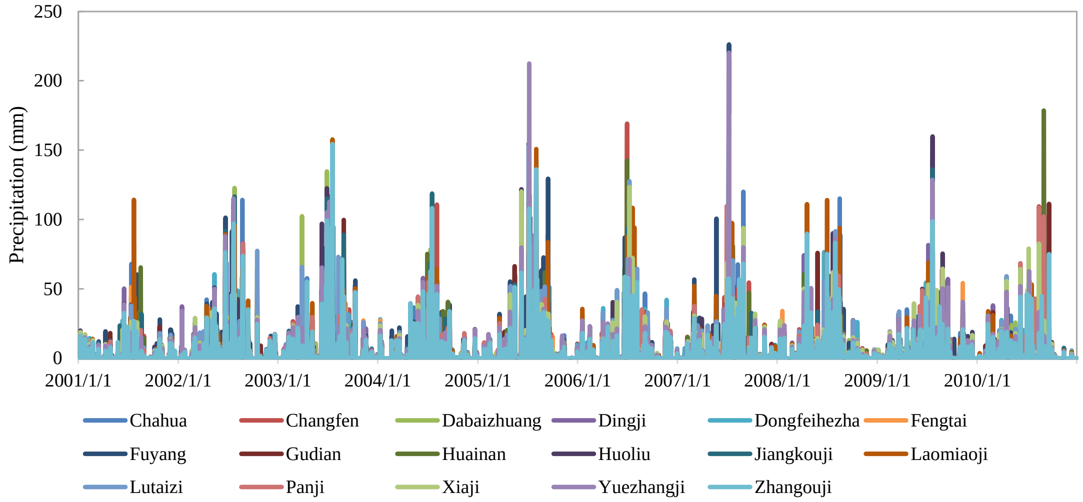

a. Daily process of precipitation in 17 stations

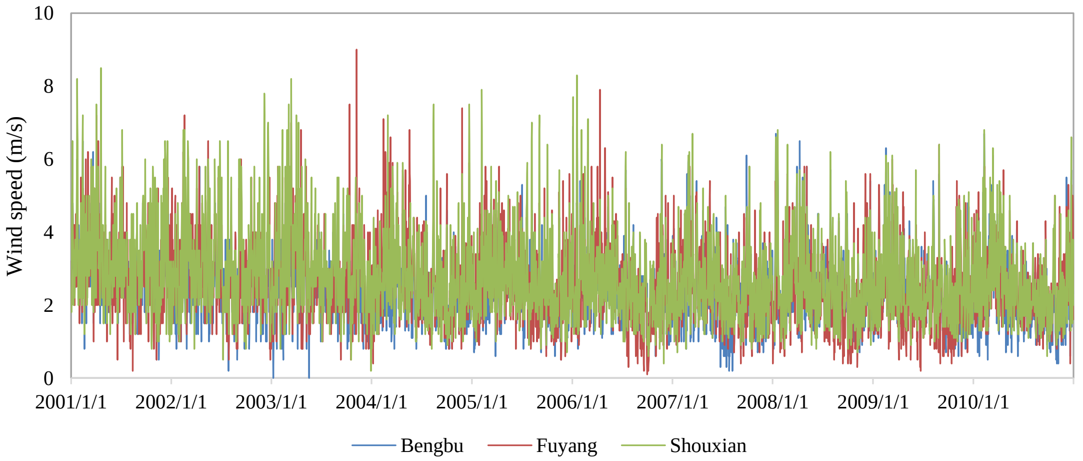

b. Daily average wind speed from 3 stations

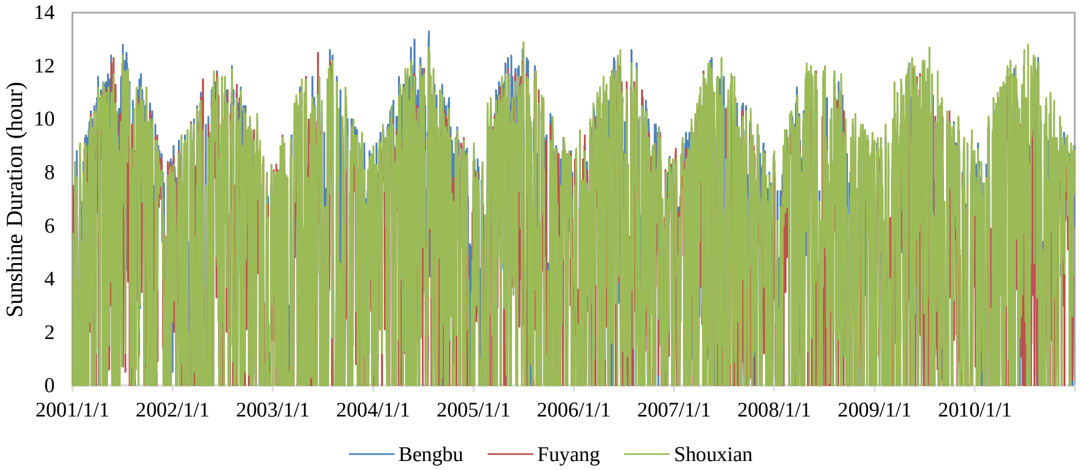

c. Daily sunshine duration of 3stations

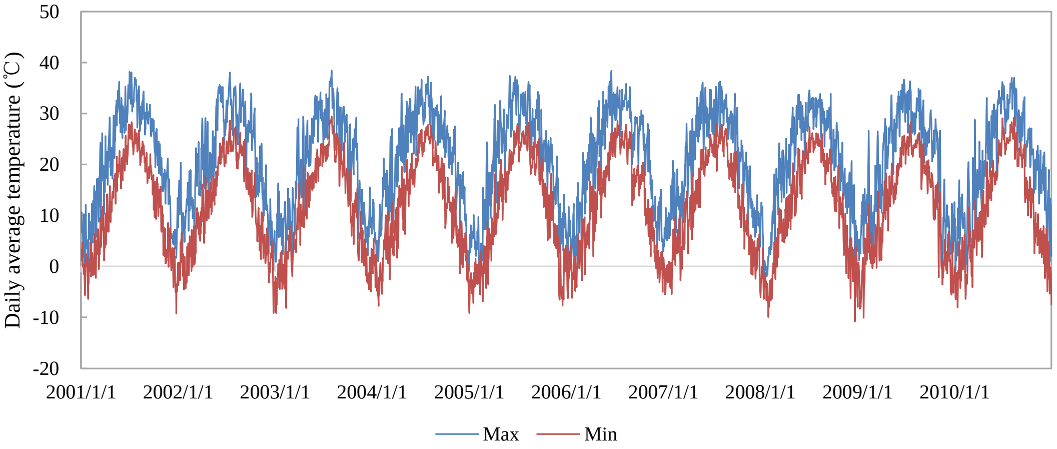

d. Maximum/Minimum air temperature of 3 stations averaged

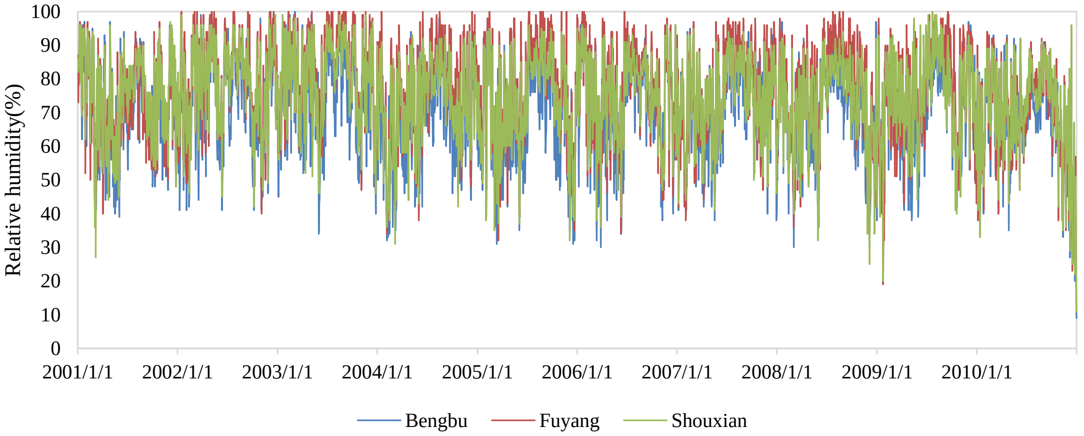

e. Daily average series of relative humidity from 3 stations

Figure S22. Weather data used in the MODCYCLE.

3.2.2 Boundary conditions of the groundwater flow model

The three rivers surrounding the study area are represented as specified head boundaries in the groundwater flow simulation, and the specified head can be

determined based on the average river stages. There are 4 river gages (Fig. S23) which have long-term continuous observed data from 1957 to 2010.

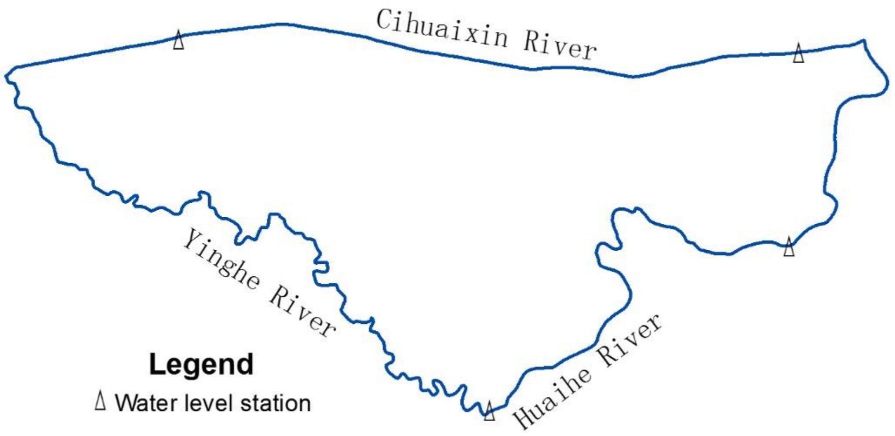

Figure S23. Location of 4 river gages surrounding the study area. Map was created using ArcGIS 9.3 (http://www.esri.com/software/arcgis/arcgis-for-desktop).

3.2.3 Crop growth period and parameters

Crop is an important participant in the hydrologic cycle of the study area. The MODCYCLE can simulate the field water moving process associated with crop growth. The growth periods and parameters of main crops are shown in Table S1.

Crop	Sowing date	Harvest date	potential heat units (℃)	Potential maximum leaf area index(-)	Maximum canopy height (m)	Maximum rooting depth (m)	Base temperature for growth (℃)	Optimal temperature for growth (℃)	Maximum stomatal conductance (m/s)
Spring soybean	May 5th	August 15th	1401	3	0.8	1.2	10	25	0.007
Cotton	April 5th	September 10th	1249	4	1	2.5	15	30	0.009
Spring corn	May 1st	August 10th	1529	5	2.5	1.2	8	25	0.007
Winter wheat	October 25th	June 5th	1989	4	0.9	1.5	0	18	0.006
Summer soybean	June 10th	September 20th	1482	3	0.8	1.2	10	25	0.007
Peanut	June 12th	September 25th	1158	4	0.5	1.2	14	27	0.006
Rice	June 10th	October 20th	1717	5	0.8	0.9	10	25	0.008
Summer corn	June 12th	September 25th	1722	5	2.5	1.2	8	25	0.007
Cole	October 15th	May 1st	1478	3	2.5	1.2	6	25	0.008

Table S1. Parameters of main crops for MODCYCLE in the study area.

3.2.4 Water usage

According to the stability of water usage, it can be divided into two types of artificial

water: agricultural irrigation water and other water use. Other water usage includes urban/rural domestic water, industrial / thermal power, and ecological/environment water usage, etc.

The applied agricultural irrigation is generally not measured or recorded. In MODCYCLE, the automatic irrigation method is used to estimate the applied agricultural irrigation. The method takes the soil moisture condition as a judgment condition, when the soil moisture in a BSU is lower than a given threshold, the model will automatically pump water from reservoir, channel, or aquifer, etc. for farmland irrigation, then complete the temporal and spatial distribution of the irrigation. Other water usage data from local water resources bulletin is distributed into subbasins according to the area of urban/rural land use distributions.

Supplementary Information 4: Other results of model calibration

The model was calibrated using river stages data from a temporary gage station and groundwater table data from 6 observation wells. In the main text, the comparison between the measured and calculated river stages and groundwater levels at an observation well is given. The comparisons at other 5 wells are presented in Fig. S24.

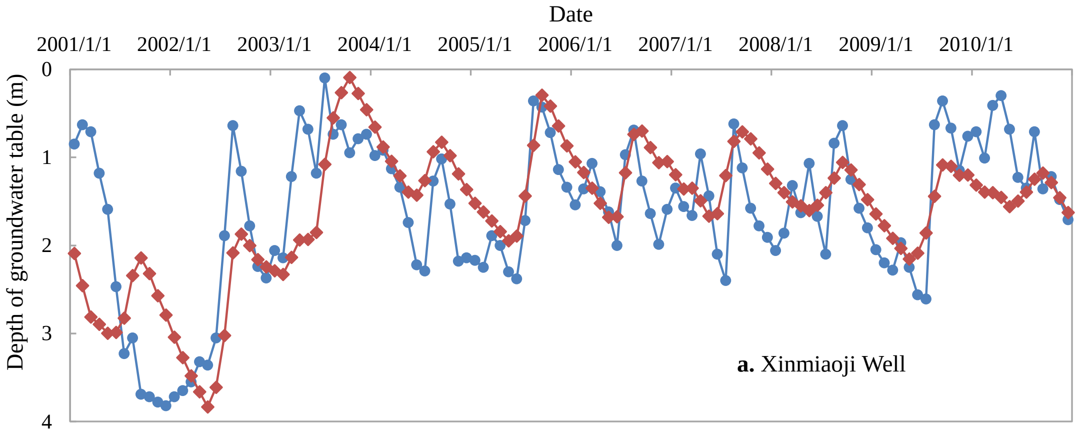

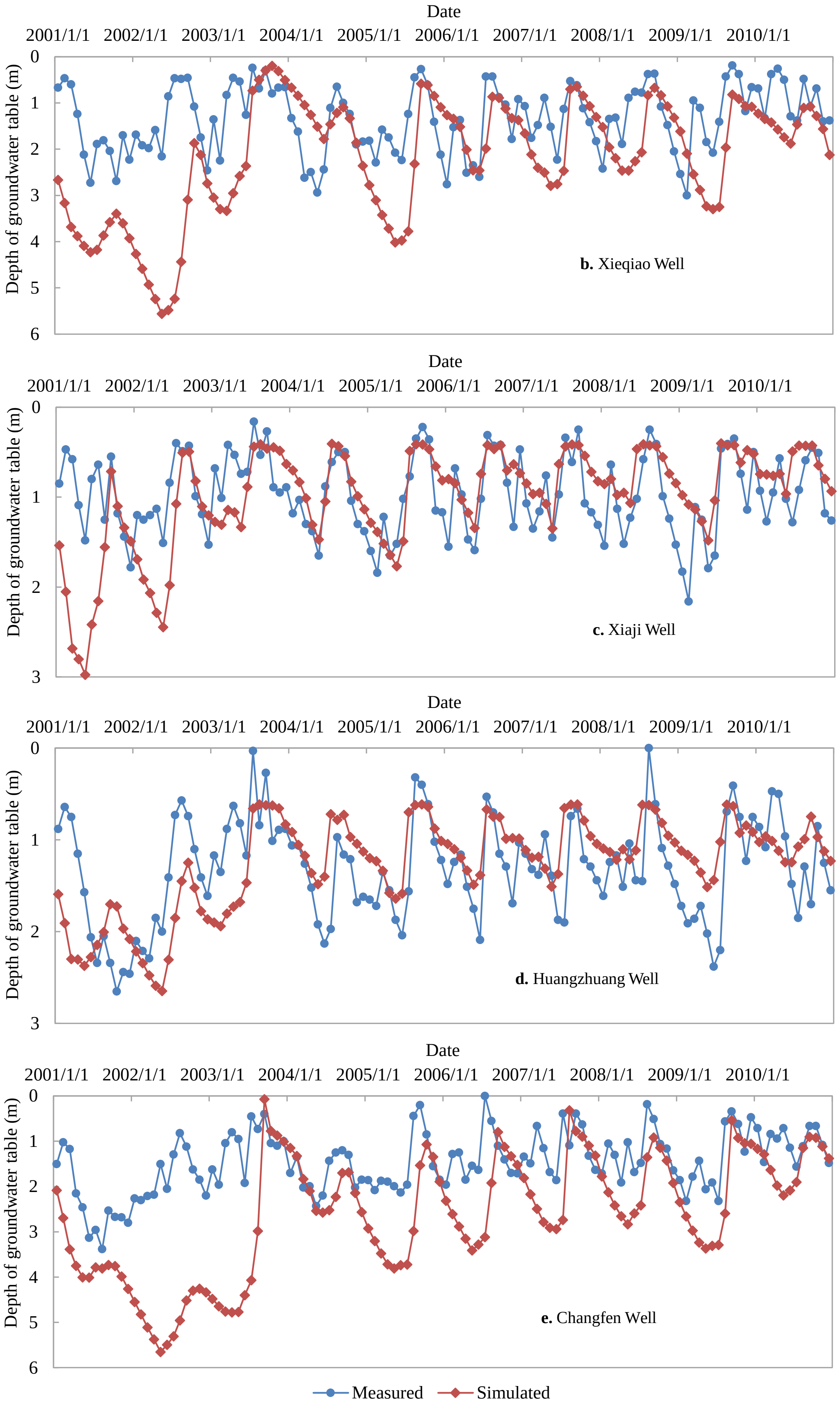

Figure S24. Comparison between the simulated and measured groundwater table depth at the (a)

Xinmiaoji, (b) Xieqiao,(c) Xiaji, (d) Huangzhuang and (e) Changfen Wells.

References

1. Alehossein, H. & Poulsen, B. Stress analysis of longwall top coal caving. *Int. J. Rock Mech. Min. Sci*. **47**, 30-41(2010).
2. Lu, C., Wang, H., Wang, J. & Xiao, W. An object oriented modularized model for basin scale water cycle simulation (in Chinese), Science Press, 2016.
3. Lu, C., Qin, D., Zhang, J. & Wang, R. MODCYCLE-an object oriented modularized hydrological model Ⅰ, theory and development (in Chinese with English abstract). *Journal of hydraulic engineering* **43**, 1135-1145(2012).
4. Lu, C., Qin, D. & Wang, H. A groundwater numerical simulation method based on the water cycle (in Chinese). Chinese Patent, No. 1535364, 2014.
5. Harbaugh, A., Banta, E., Hill, M. & McDonald, M. MODFLOW-2000, The U.S. geological survey modular groundwater model – user guide to modularization concepts and the groundwater flow process. USGS, 2000.
6. Neitsch, S. L., Arnold, J. G., Kiniry, J. R. &Williams, J. R. Soil and water assessment tool theoretical documentation(version 2005), Chapter 2.
7. Gonzalez, C. A. & Chanson, H. Experimental measurements of velocity and pressure distributions on a large broad-crested weir. *Flow Meas. Instrum.* **18**, 107 – 113(2007).
